# Association of serum HDL-cholesterol and apolipoprotein A1 levels with risk of severe SARS-CoV-2 infection

**DOI:** 10.1016/j.jlr.2021.100061

**Published:** 2021-03-02

**Authors:** James R. Hilser, Yi Han, Subarna Biswas, Janet Gukasyan, Zhiheng Cai, Ruowei Zhu, W.H. Wilson Tang, Arjun Deb, Aldons J. Lusis, Jaana A. Hartiala, Hooman Allayee

**Affiliations:** 1 Department of Preventive Medicine, University of Southern California, Los Angeles, CA, USA; 2 Department of Biochemistry and Molecular Medicine, University of Southern California, Los Angeles, CA, USA; 3 Department of Surgery, Keck School of Medicine, University of Southern California, Los Angeles, CA, USA; 4 Department of Cardiovascular and Metabolic Sciences, Lerner Research Institute, Cleveland Clinic, Cleveland OH, USA; 5 Department of Cardiovascular Medicine, Heart, Vascular and Thoracic Institute, Cleveland Clinic, Cleveland OH, USA; 6 Department of Medicine, David Geffen School of Medicine of UCLA, Los Angeles, CA, USA; 7 Department of Human Genetics, David Geffen School of Medicine of UCLA, Los Angeles, CA, USA; 8 Department of Microbiology, Immunology, and Molecular Genetics, David Geffen School of Medicine of UCLA, Los Angeles, CA, USA

**Keywords:** SAR-CoV-2, COVID-19, HDL-cholesterol, apolipoprotein A1, genetic variants, genetic risk score, Mendelian randomization, ApoE4, metabolic syndrome, ApoA1, apolipoprotein A1, CAD, coronary artery disease, 95% CI, 95% confidence interval, COVID-19, coronavirus disease 2019, GRS, genetic risk scores, ICD-10, *International Classification of Diseases, Tenth Revision*, MR, Mendelian randomization, MVMR, multivariate MR, OR, odds ratio, PC, principal component, SARS-CoV-2, severe acute respiratory syndrome coronavirus 2

## Abstract

Individuals with features of metabolic syndrome are particularly susceptible to severe acute respiratory syndrome coronavirus 2 (SARS-CoV-2), a novel coronavirus associated with the severe respiratory disease, coronavirus disease 2019 (COVID-19). Despite considerable attention dedicated to COVID-19, the link between metabolic syndrome and SARS-CoV-2 infection remains unclear. Using data from the UK Biobank, we investigated the relationship between severity of COVID-19 and metabolic syndrome-related serum biomarkers measured prior to SARS-CoV-2 infection. Logistic regression analyses were used to test biomarker levels and biomarker-associated genetic variants with SARS-CoV-2-related outcomes. Among SARS-CoV-2-positive cases and negative controls, a 10 mg/dl increase in serum HDL-cholesterol or apolipoprotein A1 levels was associated with ∼10% reduced risk of SARS-CoV-2 infection, after adjustment for age, sex, obesity, hypertension, type 2 diabetes, and coronary artery disease. Evaluation of known genetic variants for HDL-cholesterol revealed that individuals homozygous for apolipoprotein E4 alleles had ∼2- to 3-fold higher risk of SARS-CoV-2 infection or mortality from COVID-19 compared with apolipoprotein E3 homozygotes, even after adjustment for HDL-cholesterol levels. However, cumulative effects of all evaluated HDL-cholesterol-raising alleles and Mendelian randomization analyses did not reveal association of genetically higher HDL-cholesterol levels with decreased risk of SARS-CoV-2 infection. These results implicate serum HDL-cholesterol and apolipoprotein A1 levels measured prior to SAR-CoV-2 exposure as clinical risk factors for severe COVID-19 infection but do not provide evidence that genetically elevated HDL-cholesterol levels are associated with SAR-CoV-2 infection.

In December 2019, a novel virus originating from Wuhan, China was identified and associated with a rare and, in some cases, fatal form of pneumonia (1). Genetic analyses revealed the infectious agent to be a member of the enveloped single-stranded RNA family of coronaviruses, which were also the causes of previous severe acute respiratory syndrome (SARS) outbreaks in 2002 and 2003 in China and in 2012 in the Middle East (2, 3, 4, 5). Over the course of 1 year, the spread of this new coronavirus, designated as SARS-coronavirus 2 (CoV-2), has turned into a global pandemic with over 123 million reported infections and ∼2.7 million deaths as of the time of this writing (6). One intriguing aspect of SARS-CoV-2 is the considerable variability in the symptoms and manifestations exhibited by infected individuals. Clinical studies have also shown that men and individuals with preexisting metabolic conditions, such as obesity, hypertension, type 2 diabetes, chronic kidney disease, and coronary artery disease (CAD), are more prone to developing coronavirus disease 2019 (COVID-19), the severe viral pneumonia illness associated with SARS-CoV-2 infection (7, 8, 9, 10, 11, 12, 13). However, the underlying pathophysiological mechanisms for these associations are not well understood.

In addition to comorbidities, studies have also sought to identify clinical biomarkers that are associated with the severity of COVID-19 and its progression. For example, circulating levels of lipids and acute phase inflammatory proteins have been associated with poor outcomes and predictive of death among COVID-19 patients (13, 14, 15). However, these associations were based on measurements obtained at the time of symptomatic presentation or during the course of SARS-CoV-2 infection (16, 17, 18). Thus, such studies do not necessarily provide definitive evidence that the biomarkers analyzed play a causal role in influencing COVID-19 outcomes since infection itself can alter the levels of lipids and acute phase inflammatory proteins (19).

In the present study, we sought to identify metabolic disease-related clinical biomarkers using measurements made prior to the current COVID-19 outbreak and evaluate their genetic determinants for association with SARS-CoV-2 infection. We report associations between HDL-cholesterol and apolipoprotein A1 (ApoA1) levels with risk of severe SARS-CoV-2 infection. These results provide evidence for the concept that HDL metabolism may play a role in susceptibility to severe manifestations and adverse outcomes of SARS-CoV-2 infection.

## Materials and methods

### Study population

Between 2006 and 2010, the UK Biobank recruited a total of 503,325 participants who were between 40 and 69 years of age and registered with a general practitioner of the UK National Health Service (20). At enrollment, extensive data on demographics, ethnicity, education, and disease-related outcomes were obtained through questionnaires or health records. Baseline blood samples were also collected for measurement of serum biomarkers that are either established disease risk factors or routinely measured as part of clinical evaluations. Subsequent to the COVID-19 pandemic in Spring 2020, the UK Biobank began releasing the results of PCR-based tests for SARS-CoV-2 infection among its participants (Data-Field 40100). Initially, testing for SARS-CoV-2 infection in the United Kingdom was primarily restricted to symptomatic subjects admitted to one of the National Health Service hospitals (https://www.ukbiobank.ac.uk/2020/04/covid/). Most SARS-CoV-2 tests were based on combined nose/throat swabs, although lower respiratory samples may have also been analyzed for patients under intensive care settings. Thus, testing positive for SARS-CoV-2 during this period could be interpreted as being a surrogate for a severe case of COVID-19. Based on this information, we defined cases (*n* = 1,117) as symptomatic subjects who tested positive for SARS-CoV-2 infection in a hospital setting between March 16, 2020 and April 26, 2020 or whose cause of death was attributed to COVID-19 [based on *International Classification of Diseases, Tenth Revision* (ICD-10) codes U07.1 or U07.2] up until December 17, 2020. Because of the setting in which cases were identified, we defined controls as subjects who were evaluated for SARS-CoV-2 infection in inpatient or outpatient hospital settings and tested negative up until May 27, 2020 after which time community testing was begun. These criteria identified 3,544 unmatched hospital-based controls with complete demographic, clinical, and genetic data. To minimize any confounding effects from predisposing risk factors, we also defined a matched data set by matching hospital-based controls to cases at a ratio of 2:1 based on age, sex, obesity, hypertension, type 2 diabetes, and CAD using a previously described algorithm (21). These criteria defined a subset of 719 cases and 1,438 matched hospital-based controls with complete matching variables that were available for analysis. Since there would be insufficient numbers of controls of other ethnicities to allow complete matching to nonwhite European ancestry cases, we only included subjects of self-reported white European ancestry in these analyses. All UK Biobank participants provided informed consent. The study protocol was approved by the North West Multi-Centre Research Ethics Committee and carried out according to the Declaration of Helsinki principles. The present study was approved by the Institutional Review Board of the USC Keck School of Medicine.

### Statistical analyses

Differences in demographic and clinical characteristics obtained at the time of enrollment into the UK Biobank between the SAR-CoV-2-positive cases and hospital-based controls were evaluated using Chi-square tests for dichotomous/categorical variables and Mann-Whitney *U* tests for continuous traits, respectively. Differences in clinical biomarkers measured at the time of enrollment between cases and controls were evaluated using Mann-Whitney *U* tests. Spearman's correlation tests were used to assess relationships between biomarkers. HDL-cholesterol, ApoA1, and triglyceride levels were further evaluated for association with SAR-CoV-2 infection using logistic regression. For analyses with cases and unmatched hospital-based controls, the base model (model 1) included age, sex, the first 10 principal components (PC1–10), obesity, hypertension, type 2 diabetes, and CAD as covariates. Model 2 was additionally adjusted for BMI, education, and smoking. For analyses with cases and matched hospital-based controls, conditional logistic regression models were used. Model 1 was only adjusted for PC1–10 and did not include any of the matching variables (age, sex, obesity, hypertension, type 2 diabetes, and CAD), whereas model 2 was adjusted for PC1–10, BMI, education, and smoking. Education was categorized based on data provided by the UK Biobank: *A*) college/university/nursing/teaching; *B*) national vocational qualification/higher national diploma/higher national certificate; *C*) A level; *D*) O level/certificate of secondary education; or *E*) none of the above/not available. Systolic and diastolic blood pressures were based on averaging two readings taken a few minutes apart as part of the UK Biobank's clinical protocol. Hypertension status was defined based on ICD-10 code I10, self-reported hypertension, or if on hypertension medication. For subjects on hypertension medications, 10 and 5 mm Hg were added to systolic and diastolic blood pressure values, respectively (22). Asthma status was defined based on previously reported criteria (23). Type 2 diabetes status was defined based on ICD-10 code E11. CAD cases were defined as positive for ICD-10 codes I21, I22, I23, I25.2, I24.0, I24.8, I24.9, I25.0, I25.1, I25.4, I25.8, and I25.9, which included myocardial infarction and ischemic heart diseases, and Office of Population Censuses and Surveys Classification of Interventions and Procedures, version 4 codes: K40–K46, K49, K50 and K75 covering replacement, transluminal balloon angioplasty, and other therapeutic transluminal operations on coronary artery and percutaneous transluminal balloon angioplasty, and insertion of stent into coronary artery. Doctor-diagnosed and self-reported myocardial infarction was also included in the definition of CAD. Alzheimer's and dementia status was defined based on ICD-10 code F00.0, F00.1, F00.2, F00.9, F01.0, F01.1, F01.3, F01.8, F01.9, F02.0, F02.3, F02.8, F03, G30.0, G30.1, G30.8, and G30.9. All statistical analyses were carried out with SAS 9.4 (SAS Institute, Inc., Cary, NC).

### Individual variant analyses

Primary level data were used to carry out genetic analyses with 155 previously identified and independent HDL-cholesterol-associated variants (24), either individually or as a function of genetic risk scores (GRSs). Briefly, quality control of samples, DNA variants, and imputation were performed by the Wellcome Trust Centre for Human Genetics (20). From the ∼90 million SNPs imputed from the Haplotype Reference Consortium, UK10K, and 1000 Genomes imputation that were available in the UK Biobank, genotypes were extracted for the 155 HDL-cholesterol-associated SNPs. Linear regression analyses were used to test each of the 155 HDL-cholesterol SNPs individually for association with HDL-cholesterol, triglyceride (log-transformed), and LDL-cholesterol levels in up to ∼437,000 UK Biobank subjects of white European ancestry for whom these data were available, with adjustment for age, sex, PC1–10, and genotyping array. Association of the 155 HDL-cholesterol SNPs with risk of SARS-CoV-2 infection among cases and unmatched hospital-based controls was tested by logistic regression with adjustment for age, sex, PC1–10, and genotyping array. Conditional logistic regression was used for SNP analyses with cases and matched hospital-based controls with adjustment for PC1–10 and genotyping array.

For analyses with *APOE*, E3 and E4 alleles were defined based on genotypes of rs429358 and rs7412 (25) and evaluated for association with SARS-CoV-2 infection or mortality among cases. Analyses with cases and unmatched hospital-based controls were tested by logistic regression. Model 1 was adjusted for age, sex, obesity, hypertension, type 2 diabetes, CAD, BMI, education, smoking, PC1–10, and genotyping array, with additional adjustment for serum HDL-cholesterol levels in model 2. Association of ApoE4 alleles with mortality among cases only was tested using the same two logistic regression models. Conditional logistic regression was used for analyses with cases and matched hospital-based controls. Model 1 included BMI, education, smoking, PC1–10, and genotyping array as covariates (but not the matching variables), with additional adjustment for serum HDL-cholesterol levels in model 2. Linear regression for HDL-cholesterol levels and logistic regression for SARS-CoV-2 infection among cases and hospital-based controls with HDL-cholesterol-associated SNPs were performed using PLINK2 (Purcell and Chang; www.cog-genomics.org/plink/2.0/; 26). All other genetic analyses were carried out with SAS 9.4 (SAS Institute, Inc.).

### GRS analyses

In addition to single SNP analyses, we also evaluated the cumulative effect of HDL-cholesterol genetic determinants on risk of SARS-CoV-2 infection. An unweighted GRS for HDL-cholesterol was first generated for subjects by summing the number of HDL-cholesterol-raising alleles carried by the individual. We also constructed two different weighted GRS where the number of HDL-cholesterol-raising alleles carried by an individual at each locus was multiplied by the respective weight (beta) for each variant. The betas for the first GRS were derived from the linear regression analyses carried out in the ∼400,000 UK Biobank subjects with self-reported white European ancestry and summing these values across all variants. The second weighted GRS used genetic effect sizes for HDL-cholesterol levels that were previously published by Klarin *et al.* (24). The per-unit associations of the UK Biobank-based unweighted and weighted GRSs with HDL-cholesterol levels among both case-control data sets were validated by linear regression, with adjustment for age, sex, self-reported ethnicity, genotyping array, BMI, education, smoking, systolic blood pressure, type 2 diabetes, and CAD status. Logistic regression analyses were then used to evaluate the per-unit association of the unweighted and weighted GRSs with SARS-CoV-2 infection among cases and unmatched hospital-based controls as well as with mortality among cases only, with adjustment for age, sex, obesity, hypertension, type 2 diabetes, CAD, BMI, education, smoking, PC1–10, and genotyping array. Conditional logistic regression was used to test association of the three GRSs with SARS-CoV-2 infection among cases and matched hospital-based controls with adjustment for BMI, education, smoking, PC1–10, and genotyping array. GRS analyses were carried out with SAS 9.4 (SAS Institute, Inc.).

### Mendelian randomization analyses

To estimate the causal effect of genetically increased HDL-cholesterol levels on SARS-CoV-2 infection, two-sample Mendelian randomization (MR) was performed with HDL-cholesterol-associated SNPs as instrumental variables using several methods (MR Egger, weighted median, and inverse variance weighted). For these analyses, the association between HDL-cholesterol-associated SNPs and HDL-cholesterol levels in ∼400,000 UK Biobank subjects of self-reported white European ancestry was considered as the exposure variable. The association between HDL-cholesterol-related variants and risk of SARS-CoV-2 infection in the unmatched (*n* = 4,000) or matched (*n* = 2,000) hospital case-control data sets from the UK Biobank was considered as the outcome. These analyses used all HDL-cholesterol-associated variants (tier 1) that were not palindromic and for which association results for HDL-cholesterol levels and risk of SARS-CoV-2 infection were available. To minimize horizontal pleiotropy, we also carried out the same MR analyses with a subset of 35 HDL-cholesterol-associated variants (tier 2) that exhibited association with HDL-cholesterol, but not LDL-cholesterol or triglyceride, levels at the genome-wide significance threshold (*P* = 5.0 × 10^−8^). As another approach to account for pleiotropic effects, we carried out multivariate MR (MVMR) with both tier 1 and tier 2 SNPs to estimate the effect of multiple exposures (HDL-cholesterol, LDL-cholesterol, and triglycerides) simultaneously on SARS-CoV-2 infection. MVMR analyses were carried out with both tier 1 and tier 2 SNPs. Finally, MR analyses were also carried out using previously published genetic effect sizes for HDL-cholesterol levels and risk of severe SARS-CoV-2 infection (24, 27). All MR analyses were carried out in R (version 3.6.2; R Foundation for Statistical Computing) using the “TwoSampleMR” (28) and “MVMR” packages (29).

## Results

### Clinical characteristics of SARS-CoV-2 cases and controls

**Fig. 1:**
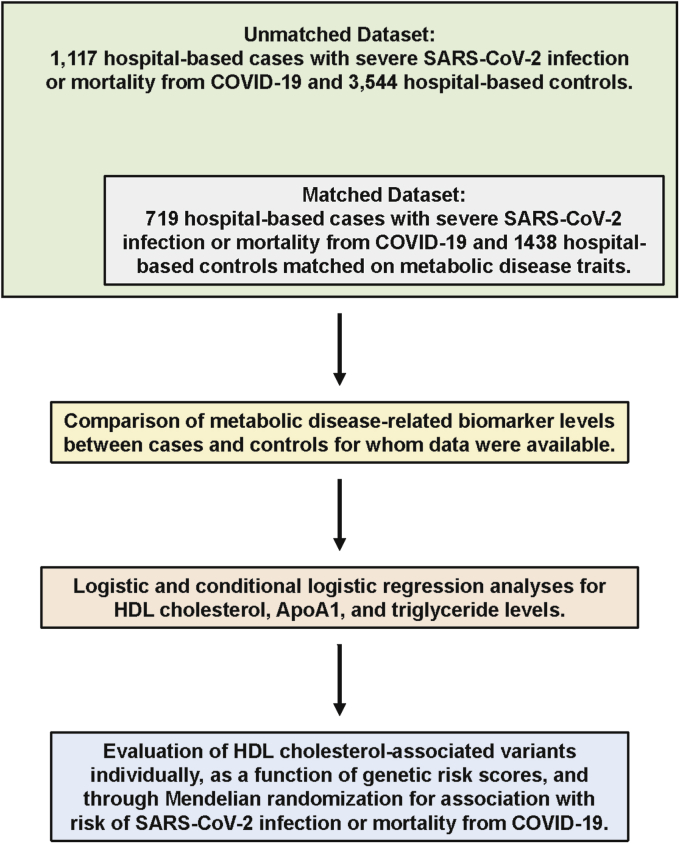
Overview of clinical and genetic analyses. A study was designed using data made available by the UK Biobank where cases were defined as symptomatic subjects who tested positive for severe acute respiratory syndrome coronavirus 2 (SARS-CoV-2) infection in a hospital setting or whose cause of death was attributed to coronavirus disease 2019 (COVID-19) (green box). Unmatched controls were defined as subjects who tested negative for SARS-CoV-2 infection in inpatient or outpatient hospital settings. A subset of these subjects was used for a data set where controls were matched to cases at a ratio of 2:1 based on complete data for age, sex, obesity, hypertension, type 2 diabetes, and coronary artery disease (gray box). Comparisons of differences in nine metabolic disease-related biomarker levels between cases and controls (yellow box) were followed by logistic regression analyses with HDL-cholesterol, ApoA1, and triglyceride levels (pink box). Previously identified genetic variants associated with HDL-cholesterol levels were then evaluated for association with risk of SARS-CoV-2 infection or mortality from COVID-19 using various analytical strategies (blue box).

Subsequent to the COVID-19 pandemic in Spring 2020, the UK Biobank began releasing the results of PCR-based tests for SARS-CoV-2 infection among its participants. Based on information related to SARS-CoV-2 infection status and other available data on demographics, clinical characteristics, serum biomarkers, and genetic variants, we defined a group of hospital-based cases and controls as well as a matched case-control data set using a subset of these subjects (Fig. 1). As a validation step, we first compared cases and unmatched controls with respect to various comorbidities that have been reported to be associated with the severity of and poor outcomes among COVID-19 patients. As shown in Table 1, SARS-CoV-2-positive cases were more likely to be male, obese, and have type 2 diabetes, elevated diastolic blood pressure, and higher BMI than unmatched hospital-based controls. These observations are largely consistent with previously reported characteristics of COVID-19 patients in other populations and further support the concept that metabolic disease increases susceptibility to developing severe symptoms of SARS-CoV-2 infection. As expected, there were no differences in clinical variables between cases and matched hospital-based controls (Table 1).

**Table 1:** Clinical characteristics of severe SARS-CoV-2 infection cases and hospital-based controls

Trait	Cases (*n* = 1,117)	Unmatched Hospital-Based Controls (*n* = 3,544)	*P*	Cases (*n* = 719)	Matched Hospital-Based Controls (*n* = 1,438)	*P*
Age at enrolment	62 (12)	60 (14)	9.4 × 10^−9^	62 (14)	62 (14)	1.00
Male/female	663/454	1,690/1,854	1.0 × 10^−11^	358/361	716/722	1.00
Education			8.8 × 10^−10^			0.06
College/university/nursing/teaching	332 (30)	1,172 (33)		238 (33)	490 (34)	
NVQ/HND/HNC (vocational qualification)	93 (8)	245 (7)		54 (8)	93 (6)	
A level (high school)	93 (8)	371 (10)		64 (9)	154 (11)	
O level/CSE (less than high school)	247 (22)	930 (26)		163 (23)	372 (26)	
None of the above/not available	352 (32)	826 (23)		200 (28)	329 (23)	
Ever smoker	665 (60)	1,872 (52)	8.6 × 10^−5^	401 (56)	747 (52)	0.09
Obesity (BMI ≥30)	405 (36)	1,049 (30)	2.8 × 10^−5^	169 (24)	338 (24)	1.00
Type 2 diabetes	191 (17)	369 (10)	2.0 × 10^−9^	32 (4)	64 (4)	1.00
Hypertension	558 (50)	1,541 (44)	1.5 × 10^−4^	287 (40)	574 (40)	1.00
Hypertension medication use	222 (20)	550 (16)	6.4 × 10^−4^	94 (13)	188 (13)	1.00
CAD	181 (16)	463 (13)	8.0 × 10^−3^	54 (8)	108 (8)	1.00
Lipid-lowering medication use^a^	350 (32)	845 (24)	5.6 × 10^−7^	162 (23)	304 (21)	0.43
Asthma	181 (16)	585 (17)	0.98	112 (16)	218 (15)	0.80
BMI	28.3 (6.5)	27.4 (6.3)	2.1 × 10^−9^	27.3 (5.1)	27.0 (5.6)	0.03
Systolic blood pressure	139.5 (28.5)	137.5 (27.0)	4.4 × 10^−3^	136.5 (28.5)	138.3 (27.0)	0.28
Diastolic blood pressure	83 (13.5)	82.5 (14.0)	0.03	82.0 (13.5)	81.5 (14.0)	0.45

CSE, certificate of secondary education; HNC, higher national certificate; HND, higher national diploma; NVQ, national vocational qualification.

Data are shown as medians and interquartile ranges for continuous variables or numbers (%) for dichotomous/categorical traits.

All cases and controls were of white European ancestry. Cases were defined as symptomatic subjects who were positive for SARS-CoV-2 infection when tested in the hospital setting or whose cause of death was due to COVID-19. Unmatched hospital-based controls were defined as UK Biobank subjects who were evaluated for SARS-CoV-2 infection in inpatient or outpatient hospital settings and tested negative. Matched hospital-based controls were a subset of the unmatched controls who were matched to cases with respect to age, sex, obesity, hypertension, type 2 diabetes, and CAD.

*P* values for differences between cases and controls were derived from Mann-Whitney *U* tests for continuous variables or Chi-square tests for dichotomous/categorical traits.

a Lipid-lowering medication use was available in 1,106 cases and 3,508 controls in the unmatched case-control data set and in 711 cases and 1,429 controls in the matched case-control data set.

### Association of clinical metabolic biomarkers with outcomes of SARS-CoV-2 infection

We next compared cases and controls for differences in serum levels of nine previously measured circulating metabolic biomarkers that are routinely evaluated clinically or are established risk factors for obesity, type 2 diabetes, hypertension, and CAD. As show in Table 2, cases had significantly lower serum HDL-cholesterol (*P* = 3.0 × 10^−11^) and ApoA1 (*P* = 5.7 × 10^−10^) levels compared with unmatched hospital-based controls. SARS-CoV-2-infected cases also had higher triglycerides, which are not unexpected given the well-known inverse relationship with HDL-cholesterol (and ApoA1) levels, and modestly higher hemoglobin A1c (Table 2). In the matched case-control data set, directionally consistent differences were also observed with HDL-cholesterol, ApoA1, and triglyceride levels (Table 2).

**Table 2:** Levels of metabolic syndrome-related biomarkers among severe SARS-CoV-2 infection cases and hospital-based controls

Biomarker	Unmatched					Matched				
	*N*	Cases	*N*	Controls	*P*	*n*	Cases	*n*	Controls	*P*
Total cholesterol (mg/dl)	1,057	209 (64)	3,367	211 (60)	0.07	676	213 (59)	1,438	212 (61)	0.64
Triglycerides (mg/dl)	1,050	150 (109)	3,365	132 (103)	2.8 × 10^−8^	670	139 (95)	1,437	129 (94)	3.1 × 10^−03^
LDL-cholesterol (mg/dl)	1,053	131 (50)	3,362	131 (47)	0.75	672	133 (45)	1,436	131 (46)	0.13
Apolipoprotein B (mg/dl)	1,048	100 (35)	3,352	99 (32)	0.53	670	101 (32)	1,434	99 (32)	0.06
HDL-cholesterol (mg/dl)	968	50 (18)	3,087	53 (19)	3.0 × 10^−11^	620	52 (18)	1,438	54 (18)	1.2 × 10^−03^
ApoA1 (mg/dl)	966	144 (35)	3,069	149 (36)	5.7 × 10^−10^	620	147 (35)	1,438	151 (35)	7.1 × 10^−04^
Lipoprotein A (mg/dl)	812	19.9 (52)	2,664	19.5 (48)	0.78	535	20.4 (55)	1,136	21.1 (47)	0.93
Glucose (mg/dl)	965	90 (15)	3,087	89.6 (14)	0.16	618	89 (14)	1,438	89 (13)	0.09
HbA1c (%)	1,064	5.5 (0.5)	3,355	5.4 (0.5)	4.1 × 10^−8^	682	5.4 (0.5)	1,347	5.4 (0.5)	0.49

HbA1c, hemoglobin A1c.

Data are shown as medians and interquartile ranges in cases and controls for whom serum biomarker levels were available.

*P* values for differences between cases and controls were derived from Mann-Whitney *U* tests.

**Fig. 2:**
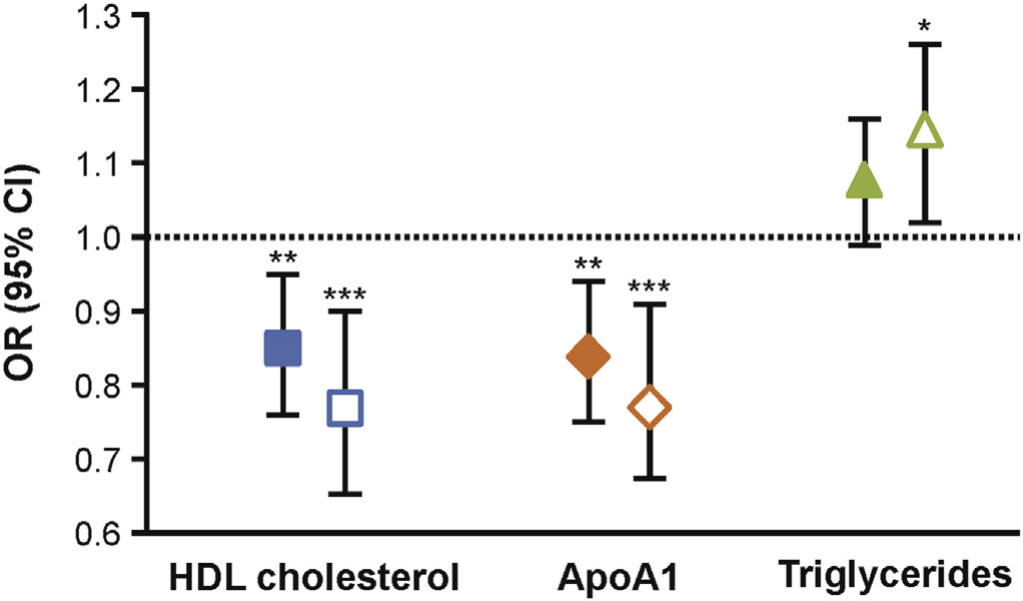
Association of serum HDL-cholesterol, apolipoprotein A1 (ApoA1), and triglyceride levels with risk of severe severe acute respiratory syndrome coronavirus 2 (SARS-CoV-2) infection. Increased levels of HDL-cholesterol or ApoA1 were associated with ∼15–20% decreased risk of SARS-CoV-2 infection among unmatched (filled-in symbols) or matched (open symbols) cases and controls, even after adjustment for multiple covariates. Increased triglyceride levels were associated with ∼10–15% increased risk of SARS-CoV-2 infection in only the matched case-control data set. Logistic regression analyses were used for comparisons between cases and unmatched controls with adjustment for age, sex, principal component 1–10, obesity, hypertension, type 2 diabetes, CAD, BMI, education, and smoking. Conditional logistic regression analyses were used for comparisons between cases and matched controls with adjustment for only principal component 1–10, BMI, education, and smoking. Data are shown as odds ratios (ORs) with 95% confidence intervals (95% CIs) for a one unit increase in the interquartile range of each biomarker (provided in Table 2). ∗*P* < 0.05; ∗∗*P* < 0.01; and ∗∗∗*P* < 0.005.

We next focused on HDL-cholesterol, ApoA1, and triglycerides since these biomarkers are metabolically related, have been shown to be relatively stable during adulthood (30), and were associated with SARS-CoV-2 infection, even after a conservative Bonferroni-adjusted *P* value for testing nine biomarkers (*P* = 0.05/9 = 0.006) (Table 2). In comparisons between cases and unmatched hospital-based controls, a 10 mg/dl or one interquartile range increase in serum HDL-cholesterol levels was associated with significantly (*P* = 4.0 × 10^−04^) reduced risk of SARS-CoV-2 infection [odds ratio (OR), 0.90; 95% confidence interval (95% CI), 0.85–0.95 and OR, 0.82; 95% CI, 0.73–0.91, respectively] (Table 3). The effect sizes of these protective associations were similar and only slightly decreased in significance after further adjustment for BMI, education, and smoking (Table 3 and Fig. 2). Similar associations were observed between high ApoA1 levels and reduced risk of SARS-CoV-2 infection (Table 3 and Fig. 2). Comparisons among cases and matched hospital-based controls also yielded equivalent protective associations with HDL-cholesterol and ApoA1 levels (Table 3 and Fig. 2). However, association of serum triglyceride levels with SARS-CoV-2 infection only remained significant in the matched case-control data set in the fully adjusted model (Table 3 and Fig. 2). In addition, the associations of HDL-cholesterol, ApoA1, and triglyceride levels with SARS-CoV-2 infection were not driven by cases whose cause of death was due to COVID-19 since exclusion of these subjects yielded associations that were similar in magnitude and significance to those that included all cases (supplemental Table S1). Taken together, these results indicate that elevated HDL-cholesterol and ApoA1 levels based on measurements that were made prior to SARS-CoV-2 exposure are inversely associated with risk of severe SARS-CoV-2 infection among subjects from the UK Biobank.

**Table 3:** Association of serum HDL-cholesterol, ApoA1, triglyceride levels with risk of severe SARS-CoV-2 infection

Trait	Cases versus Unmatched Hospital-Based Controls^a^				Cases versus Matched Hospital-Based Controls^b^			
	*n*	Per 10 mg/dl	Per IQR	*P*	*n*	Per 10 mg/dl	Per IQR	*P*
		OR (95% CI)	OR (95% CI)			OR (95% CI)	OR (95% CI)	
HDL-Cholesterol								
Model 1	968/3,087	0.90 (0.85–0.95)	0.82 (0.73–0.91)	4.0 × 10^−04^	620/1,240	0.85 (0.78–0.92)	0.74 (0.63–0.86)	1.1 × 10^−04^
Model 2	968/3,087	0.92 (0.86–0.97)	0.85 (0.76–0.95)	5.1 × 10^−03^	620/1,240	0.87 (0.79–0.94)	0.77 (0.65–0.90)	1.2 × 10^−03^
ApoA1								
Model 1	966/3,069	0.94 (0.92–0.97)	0.82 (0.73–0.91)	3.0 × 10^−04^	620/1,240	0.93 (0.89–0.96)	0.76 (0.66–0.88)	2.2 × 10^−04^
Model 2	966/3,069	0.95 (0.92–0.98)	0.84 (0.75–0.94)	9.0 × 10^−03^	620/1,240	0.93 (0.89–0.97)	0.78 (0.67–0.91)	1.2 × 10^−03^
Triglycerides								
Model 1	1,050/3,365	1.01 (1.00–1.02)	1.09 (1.01–1.19)	0.03	670/1,339	1.02 (1.00–1.03)	1.15 (1.04–1.28)	7.5 × 10^−03^
Model 2	1,050/3,365	1.01 (1.00–1.01)	1.07 (0.99–1.16)	0.10	670/1,339	1.01 (1.00–1.03)	1.14 (1.02–1.26)	0.02

IQR, interquartile range.

Sample sizes (*n*) indicate number of cases/controls. Data are shown as ORs and 95% CIs.

a Logistic regression analysis: model 1 was adjusted for age, sex, PC1–10, obesity, hypertension, type 2 diabetes, and CAD. Model 2 included the same covariates as model 1 with additional adjustment for BMI, education, and smoking.

b Conditional logistic regression analysis: model 1 was only adjusted for PC1–10 and did not include any of the matching variables (age, sex, obesity, hypertension, type 2 diabetes, and CAD). Model 2 was adjusted for PC1–10, BMI, education, and smoking.

### Evaluation of genetic determinants of HDL-cholesterol with SARS-CoV-2 infection

We next used a genetics strategy to determine whether the associations between HDL-cholesterol and ApoA1 levels and SARS-CoV-2 infection represented causal relationships. Given the high correlation between HDL-cholesterol and ApoA1 in the case-control data sets (*r* = 0.92; *P* < 1.0 × 10^−300^), we focused these analyses on only HDL-cholesterol. As a first step, we selected independent lead SNPs at 155 previously identified loci for HDL-cholesterol levels (24) and validated them in the ∼400,000 UK Biobank subjects of self-reported white European ancestry for whom HDL-cholesterol levels were available. Of the 155 SNPs, nearly all variants yielded directionally consistent associations with HDL-cholesterol levels at *P* < 0.05, and all but 19 exceeded a Bonferroni-corrected significance threshold (*P* = 0.05/155 = 3.2 × 10^−4^) (supplemental Table S2). Consistent with previous studies (24), many of these variants were also associated with serum triglyceride and/or LDL-cholesterol levels, although the significance of the association signals was nearly all attenuated compared with HDL-cholesterol levels (supplemental Table S2). We next tested the 155 HDL-cholesterol variants individually for association with SARS-CoV-2 infection (Table S2). Nine variants yielded *P* < 0.05 in comparisons between cases and unmatched and/or matched hospital-based controls, although none would be considered significant at a Bonferroni-corrected threshold (supplemental Table S3).

Of the variants associated with SARS-CoV-2 infection in either case-control data set, rs429358 yielded the strongest association among unmatched cases and control (*P* = 4.6 × 10^−4^) (Table 3) and was one of the most significantly associated variants with HDL-cholesterol levels (*P* = 6.9 × 10^−142^) (Table 2). Rs429358 is a missense variant in *APOE* and used in combination with another amino acid substitution (rs7412) to define three common alleles of *APOE* (E2, E3, and E4). ApoE2 and ApoE4 alleles are known to increase lipid levels, and ApoE4 is further recognized as the strongest known genetic risk factor for Alzheimer's disease (31, 32). Recent analyses have also shown that homozygosity for ApoE4 was associated with significantly higher risk of SARS-CoV-2 infection as well as mortality from COVID-19, even after exclusion of subjects with potential confounding comorbidities, such as dementia, hypertension, type 2 diabetes, or CAD (33, 34). Based on these observations, we evaluated whether previously reported associations between E4/E4 genotype and SARS-CoV-2-related outcomes could be due to effects on HDL-cholesterol levels. Consistent with previous observations (33, 34), ApoE4 homozygotes had 2- to 3-fold higher risk of SARS-CoV-2-related outcomes compared with ApoE3/ApoE3 (Table 4). However, adjustment for serum HDL-cholesterol levels did not attenuate the effect size or significance of the associations between ApoE4/ApoE4 genotype and SARS-CoV-2 infection among the unmatched (OR, 2.27; 95% CI, 1.56–3.30; *P* = 1.6 × 10^−5^) and matched (OR, 3.15; 95% CI, 1.75–5.68; *P* = 1.4 × 10^−4^) data sets or mortality because of COVID-19 among the cases (OR, 2.57; 95% CI, 1.31–5.05; *P* = 6.0 × 10^−3^) (Table 4). Exclusion of subjects with dementia or Alzheimer's disease also yielded directionally consistent, but slightly attenuated, association of ApoE4/ApoE4 genotype with SARS-CoV-2-related outcomes after adjustment for HDL-cholesterol levels (supplementarl Table S4), suggesting these results were not confounded by Alzheimer's disease or dementia.

**Table 4:** Association of ApoE4 alleles with severe SARS-CoV-2 infection and mortality from COVID-19 is independent of serum HDL-cholesterol levels

Comparison Groups	ApoE Genotypes			*P*^a^
	E3/E3	E3/E4	E4/E4	
Cases versus unmatched hospital-based controls (*n*)^b^	531/1,763	249/770	51/79	
Model 1	1	1.12 (0.94–1.34)	2.23 (1.58–3.32)	1.3 × 10^−5^
Model 2	1	1.11 (0.93–1.33)	2.27 (1.56–3.30)	1.6 × 10^−5^
Cases versus matched hospital-based controls (*n*)^c^	342/636	144/242	32/23	
Model 1	1	1.22 (0.94–1.57)	3.15 (1.75–5.68)	1.4 × 10^−4^
Model 2	1	1.23 (0.95–1.60)	3.15 (1.75–5.68)	1.4 × 10^−4^
Mortality among cases (yes/no)^b^	236/295	124/125	32/19	
Model 1	1	1.22 (0.85–1.73)	2.55 (1.30–5.00)	6.6 × 10^−3^
Model 2	1	1.21 (0.85–1.72)	2.57 (1.31–5.05)	6.0 × 10^−3^

Data are shown as ORs and 95% CI.

a *P* values are for comparisons between E4/E4 versus E3/E3 groups.

b Logistic regression analysis: model 1 was adjusted for age, sex, PC1–10, obesity, hypertension, type 2 diabetes, CAD, BMI, education, smoking, and genotyping array. Model 2 was adjusted for the same variables as model 1 with additional adjustment for serum HDL-cholesterol levels.

c Conditional logistic regression analysis: model 1 was adjusted for PC1–10, BMI, education, smoking, and genotyping array. Model 2 was adjusted for the same variables as model 1 with additional adjustment for serum HDL-cholesterol levels.

Finally, we evaluated the cumulative genetic effects of HDL-cholesterol-raising alleles with SARS-CoV-2-related outcomes. Among cases and unmatched hospital-based controls, a one unit increase in an unweighted or weighted GRS with HDL-cholesterol-raising alleles was associated with a 0.31 mg/dl (*P* < 0.0001) or 0.90 mg/dl (*P* < 0.0001) increase in HDL-cholesterol levels, respectively, in the fully adjusted model. When analyzing cases and matched hospital-based controls, a one unit increase in the unweighted or weighted HDL-cholesterol GRS was similarly associated with a 0.27 mg/dl (*P* < 0.0001) or 0.79 mg/dl (*P* < 0.0001) increase in HDL-cholesterol levels, respectively, in the fully adjusted model. These results thus validate the cumulative genetic effects of HDL-cholesterol-raising alleles on HDL-cholesterol levels in both cases of control data sets. We next tested the unweighted and weighted GRSs for association with SARS-CoV-2 infection or mortality because of COVID-19. As shown in supplemental Table S5, there was no evidence for association of the unweighted or weighted HDL-cholesterol GRSs with either of these outcomes. Evaluation of cumulative genetic burden using previously published effect sizes for the 155 SNPs (24) also did not provide evidence for association of a weighted HDL-cholesterol GRS with SARS-CoV-2 infection (supplemental Table S5). We also used MR to evaluate whether genetically increased HDL-cholesterol levels were causally associated with SARS-CoV-2-related outcomes. These analyses, which included two-sample MR and a multivariate approach (MVMR) to account for the pleiotropic association of HDL-cholesterol variants with other lipid traits, did not yield evidence that genetically elevated HDL-cholesterol levels were associated with decreased risk of SARS-CoV-2 infection or mortality because of COVID-19 (supplemental Table S6). MR analyses carried out with previously published effect sizes for HDL-cholesterol and risk of severe SARS-CoV-2 infection (24, 27) yielded similar results (supplemental Table S6).

## Discussion

In the present study, we sought to identify circulating metabolic biomarkers that were associated with risk of SARS-CoV-2 infection and its adverse outcomes using measurements obtained at the time of enrollment into the UK Biobank but in the context of the current COVID-19 pandemic. Of the biomarkers evaluated, protective associations were observed between SARS-CoV-2 infection and high levels of HDL-cholesterol or ApoA1, particularly among individuals with the highest levels that were one interquartile range (∼20 mg/dl) above the median in whom risk was reduced by ∼20%. Importantly, the effect sizes of the protective associations observed between HDL-cholesterol or ApoA1 levels and SARS-CoV-2 infection among cases and matched controls were nearly identical to those obtained in analyses using unmatched controls. These results suggest that the associations observed with HDL-cholesterol or APOA1 levels are not likely confounded by preexisting conditions known to increase susceptibility to COVID-19, such as obesity and type 2 diabetes, since these factors were used as matching variables. In this regard, it is noteworthy that a recent study demonstrated the association of genetically higher BMI and lifetime smoking with increased risk of severe COVID-19 (35). Moreover, our findings are consistent with other studies demonstrating similarly pronounced inverse associations between HDL-cholesterol levels and SARS-CoV-2 infection (36), other types of viral infections in the general population (37), and increased risk of death among patients with low HDL-cholesterol and ApoA1 levels that have been admitted to intensive care units (38, 39).

Our observations raise important questions with respect to the underlying biological mechanisms through which HDL-cholesterol and ApoA1 protect against infection by SARS-CoV-2 (and other pathogens). Notably, HDL-cholesterol is now recognized as having important immune-mediating properties and other functions beyond its critical role in reverse cholesterol transport (40, 41, 42, 43). For example, HDL-cholesterol can protect against infections by a variety of pathogens, including bacteria and parasites, and can directly bind and neutralize various DNA and RNA viruses through ApoA1-mediated inhibition of viral fusion and entry into host cells (44, 45, 46, 47, 48). It would therefore be important to determine whether HDL-cholesterol possesses the same antiviral activity against SARS-CoV-2 since this could provide one explanation for the association of high HDL-cholesterol and ApoA1 with decreased risk of symptomatic SARS-CoV-2 infection. Furthermore, one major life-threatening complication of COVID-19 is systemic coagulopathy (49), and HDL-cholesterol has been shown to mediate various aspects of thrombosis and platelet activation as well (50). Thus, the wide array of functional properties displayed by HDL-cholesterol, which could potentially be independent of circulating levels, provide multiple mechanisms through which this lipoprotein could modulate both the initial phase of SARS-CoV-2 infection as well as its adverse clinical sequelae. However, validation of the clinical associations observed in the present study will still require direct experimentation.

Another important aspect of our study is that nearly all currently available biomarker measurements in the UK Biobank were made a decade or more prior to the emergence of SARS-CoV-2. Therefore, the associations we observe with SARS-CoV-2 infection are based on the assumption that the levels of HDL-cholesterol and ApoA1 levels do not change over time among middle-aged adults or are not strongly affected by environmental factors. This notion is supported by analyses with the Framingham Heart Study in which HDL-cholesterol levels were shown to be stable over the adult life course and across a spectrum of values (30). In this regard, the HDL-cholesterol (and ApoA1) values obtained in UK Biobank subjects prior to the SARS-CoV-2 outbreak could be inferred as a reasonable reflection of current serum levels. Based on this notion, our observed clinical associations would suggest a potential causal relationship between HDL-cholesterol and SARS-CoV-2 infection. However, we did not observe evidence that genetically higher HDL-cholesterol levels were associated with SARS-CoV-2 infection. By comparison, a recently published analysis with >400,000 subjects did provide evidence for a causal relationship between HDL-cholesterol levels and risk of non-COVID-19-related infectious hospitalizations (51), although the protective genetic associations in that study were very modest (∼4% reduced risk) compared with the ∼40% decreased risk observed clinically with HDL-cholesterol levels. Another consideration is that nearly all previously identified genetic variants for HDL-cholesterol levels have, to some degree, pleiotropic associations with other lipid or metabolic traits (24). This may be particularly relevant for the *APOE* variants and isoforms we tested, which have effects on multiple lipid fractions and may thus not be suitable instruments for inferring causality about the role of HDL in SARS-CoV-2 infection. As discussed recently, such horizontal pleiotropy may lead to false-positive causal associations but can also decrease the power for detecting true positive associations through the introduction of noise (52). Interestingly, these potential limitations were recently addressed with respect to HDL-cholesterol as a causal biomarker of CAD (53). By using MVMR analysis to account for pleiotropy and CAD case-control data sets that had ∼5-fold larger sample sizes compared with those used in the previous studies (54), these authors provided evidence for HDL-cholesterol levels being independently associated with CAD through locus- and mechanism-specific causal effects. However, despite using a similar approach in our study, the sample sizes in our MR analyses likely did not provide sufficient power to detect associations between genetically increased HDL-cholesterol levels and SARS-CoV-2 infection. Thus, we cannot rule out the presence of a causal association between elevated HDL-cholesterol levels and risk of COVID-19 based on the data sets used in the present study, which will need to be addressed in larger studies.

As part of our genetic analyses, we also evaluated ApoE4 alleles for associations with SARS-CoV-2 outcomes based on recent analyses in the UK Biobank (33, 34). Although ApoE is found on certain HDL subfractions where it facilitates reverse cholesterol transport, our results suggest that ApoE4 affects susceptibility to severe SARS-CoV-2 infection and mortality from COVID-19 independently of HDL-cholesterol levels. This concept is supported by previous studies showing that ApoE has other biological functions that could be relevant to SARS-CoV-2 infection (55). For example, ApoE has been shown to prevent infection of various viruses, similar to HDL and ApoA1 (56), with ApoE4 having less effective antiviral activity compared with ApoE3 (57). ApoE can also decrease coagulation by binding to heparin sulfate proteoglycans that are expressed on endothelial cells (50). Thus, the similarity of functional properties of ApoEs to those of HDL also provides plausible biological mechanisms for the genetic associations we observed between ApoE4 and SARS-CoV-2 infection outcomes.

While our results point to clinical risk factors for SARS-CoV-2-related outcomes, certain limitations of our study still need consideration. First, depending on the outcome being investigated, defining cases and an appropriate control group using data from the UK Biobank may be challenging, particularly given the evolving nature of the COVID-19 pandemic and improvements in patient care. We attempted to address this potential issue by using a relatively strict definition of cases as subjects who displayed severe COVID-19 symptoms and tested positive for SARS-CoV-2 in a hospital setting. Such individuals could be viewed as severe COVID-19 cases based on information released by the UK Biobank (https://www.ukbiobank.ac.uk/2020/04/covid/). We also sought to best define controls as subjects who tested negative for SARS-CoV-2 in the same hospital-based inpatient or outpatient settings. In order to minimize additional potential confounding effects in the analyses, a subset of the controls was also matched to cases with respect to known COVID-19-predisposing comorbidities. It is still possible that other unidentified diseases could have confounded the association between clinically measured HDL-cholesterol levels and SARS-CoV-2 infection. However, the consistency of the results obtained within the framework of our study design, coupled with extensive prior biological evidence that HDL and ApoA1 possess antiviral activity, suggests that the clinical relationships we observed with SARS-CoV-2 infection outcomes represent true associations. Furthermore, despite prior studies showing the relative stability of HDL-cholesterol and ApoA1 concentrations over time, levels of these biomarkers measured at the time of enrollment into the UK Biobank may not be good indications of present day values. Finally, we did not independently replicate the associations of HDL-cholesterol and ApoA1 levels and *APOE* genotype with SARS-CoV-2 outcomes, which will need to be addressed in additional data sets.

In summary, we identified HDL-cholesterol and ApoA1 levels as clinical risk factors for SARS-CoV-2 infection. Importantly, plausible explanations for these associations can be inferred based on our rapidly expanding knowledge of the pathology caused by SARS-CoV-2 as well as the known biological functions of HDL-cholesterol and ApoA1. The results of our study thus provide new avenues for understanding the progression of COVID-19 and additional pathways to evaluate for potential diagnostic and/or therapeutic development.

## Data availability

Individual-level data used in the present study are available upon application to the UK Biobank (https://www.ukbiobank.ac.uk/). All other relevant data are available upon request from the authors.

## Supplemental data

This article contains supplemental data (24, 27).

## Conflict of interest

The authors declare that they have no conflicts of interest with the contents of this article.
