# Supplementary material for: Association of serum HDL-cholesterol and apolipoprotein A1 levels with risk of severe SARS-CoV-2 infection
Source: J Lipid Res. 2021 Mar 2;62:100061. doi: 10.1016/j.jlr.2021.100061 (PMC7923911; doi:10.1016/j.jlr.2021.100061)
Supplement: Supplemental Tables — S1 to S6 [file mmc1.pdf]

Table S1. Association of Serum HDL cholesterol, ApoA1, Triglyceride Levels with Risk of Severe SARS-CoV-2 Infection After Exclusion of Subjects who Died from COVID-19.

IQR=interquartile range.

Sample sizes (n) indicate number of cases/controls. Data are shown as odds ratios (OR) and 95% confidence intervals (CI).

[a]Logistic regression analysis: Model 1 was adjusted for age, sex, PC1-10, obesity, hypertension, type 2 diabetes, and CAD. Model 2 included the same covariates as model 1 with additional adjustment for BMI, education, and smoking.

[b]Conditional logistic regression analysis: Model 1 was only adjusted for PC1-10 and did not include any of the matching variables (age, sex, obesity, hypertension, type 2 diabetes, and CAD). Model 2 was adjusted for PC1-10, BMI, education, and smoking.

	[a]Cases vs. Unmatched Hospital-based Controls				[b]Cases vs. Matched Hospital-based Controls			
**Trait**		**Per 10mg/dL**	**Per IQR**			**Per 10mg/dL**	**Per IQR**	
**HDL cholesterol**	**n**	**OR (95% CI)**	**OR (95% CI)**	**P-value**	**n**	**OR (95% CI)**	**OR (95% CI)**	**P-value**
Model 1	502/3087	0.88 (0.82 - 0.96)	0.79 (0.68 - 0.92)	2.0E-04	345/690	0.84 (0.75 - 0.95)	0.73 (0.60 - 0.90)	3.4E-03
Model 2	502/3087	0.90 (0.83 - 0.98)	0.82 (0.71 - 0.96)	0.01	345/690	0.86 (0.76 - 0.96)	0.75 (0.60 - 0.94)	0.01
**ApoA1**								
Model 1	502/3069	0.95 (0.91 - 0.99)	0.82 (0.71 - 0.95)	8.0E-03	346/692	0.93 (0.88 - 0.98)	0.78 (0.64 - 0.94)	0.01
Model 2	502/3069	0.96 (0.92 - 0.99)	0.85 (0.74 - 0.99)	0.03	346/692	0.93 (0.88 - 0.99)	0.79 (0.64 - 0.97)	0.02
**Triglycerides**								
Model 1	545/3365	1.01 (1.00 - 1.02)	1.11 (1.00 - 1.23)	0.04	373/745	1.02 (1.01 - 1.04)	1.25 (1.07 - 1.44)	3.7E-03
Model 2	545/3365	1.01 (1.00 - 1.02)	1.09 (0.98 - 1.21)	0.10	373/745	1.02 (1.01 - 1.04)	1.23 (1.06 - 1.42)	7.5E-03

Table S2. Association of 155 Previously Reported HDL Cholesterol SNPs with Lipid Levels in the UK Biobank.

EA, effect (HDL cholesterol raising) allele; NEA, non-effect allele.

Linear regression analyses were carried out in subjects of self-reported white European ancestry for whom each lipid trait was available, with adjustment for age, sex, PC1-10, and genotyping array.

[a]Based on log-transformed values.

[b]Proxy for previously reported variant.

[c]Excluded from tier 1 in MR analyses due to being either palindromic (rs4655268 and rs4766578) or missing association results for SARS-CoV-2 infection (rs77375493, rs138326449, rs139271800).

[d]Considered as tier 2 SNP in MR analyses (see Table S6).

						HDL cholesterol (mg/dL)				Triglycerides (mg/dL)				LDL cholesterol (mg/dL)			
SNP	CHR	BP (hg19)	EA	NEA	EAF	N	BETA	SE	P-value	N	[a]BETA	[a]SE	[a]P-Value	N	BETA	SE	P-value
rs79598313	1	27,284,913	C	T	0.98	400,515	1.055	0.099	2.8E-26	437,275	-0.042	0.004	6.6E-32	436,811	-1.835	0.237	8.9E-15
rs3768321	1	40,035,928	G	T	0.81	399,400	0.682	0.038	1.9E-72	436,051	-0.014	0.001	6.8E-25	435,587	0.116	0.090	2.0E-01
[b]rs11207977	1	62,977,307	C	T	0.65	400,620	0.193	0.032	9.8E-10	437,382	0.041	0.001	2.1E-292	436,919	1.340	0.075	1.7E-71
[d]rs10874777	1	93,862,020	T	C	0.37	397,552	0.307	0.031	8.3E-23	434,036	-0.006	0.001	1.9E-08	433,575	0.202	0.074	6.5E-03
rs2878349	1	107,549,245	G	A	0.67	400,068	0.056	0.032	0.08	436,803	0.001	0.001	0.60	436,342	-0.424	0.076	2.4E-08
rs3832016	1	109,818,158	C	CT	0.22	400,524	0.426	0.037	2.5E-31	437,281	-0.005	0.001	8.7E-05	436,818	-3.948	0.087	7.9E-450
rs10494363	1	149,909,495	A	G	0.08	400,323	0.364	0.056	7.9E-11	437,066	-0.014	0.002	7.9E-12	436,603	-0.081	0.133	5.4E-01
[d]rs267738	1	150,940,625	G	T	0.22	400,718	0.309	0.036	1.7E-17	437,493	0.000	0.001	0.88	437,029	-0.283	0.086	1.0E-03
[d]rs4077194	1	178,533,832	G	T	0.47	400,074	0.256	0.030	2.0E-17	436,795	-0.005	0.001	1.0E-06	436,333	0.157	0.072	2.8E-02
[d]rs61805076	1	182,154,990	T	C	0.67	398,982	0.391	0.032	1.6E-34	435,594	-0.002	0.001	0.08	435,134	-0.369	0.076	1.2E-06
rs10911505	1	184,049,978	T	C	0.64	397,843	0.086	0.031	6.1E-03	434,334	-0.002	0.001	0.04	433,873	0.167	0.075	2.5E-02
[d]rs823114	1	205,719,532	A	G	0.54	400,104	0.183	0.030	1.3E-09	436,816	-0.002	0.001	0.049	436,353	0.061	0.072	4.0E-01
[c]rs4655268	1	214,992,980	C	G	0.45	382,884	0.063	0.031	0.04	418,057	-0.001	0.001	0.43	417,618	0.156	0.073	3.4E-02
rs2066152	1	219,664,030	A	G	0.39	394,334	0.208	0.031	1.8E-11	430,489	-0.011	0.001	3.6E-22	430,034	-0.155	0.074	0.04
rs2642438	1	220,970,028	G	A	0.71	400,718	0.379	0.033	8.6E-31	437,493	-0.006	0.001	3.2E-08	437,029	0.845	0.078	3.2E-27
rs4846914	1	230,295,691	A	G	0.60	400,718	0.866	0.031	1.6E-174	437,493	-0.025	0.001	3.0E-113	437,029	-0.221	0.073	2.6E-03
[d]rs6710091	2	239,597	G	C	0.35	400,262	0.200	0.032	2.3E-10	436,991	-0.005	0.001	3.1E-05	436,527	0.137	0.075	6.9E-02
[d]rs4850047	2	3,634,753	T	C	0.13	400,718	0.359	0.044	6.0E-16	437,493	-0.009	0.002	2.0E-08	437,029	0.645	0.106	1.0E-09
rs676210	2	21,231,524	A	G	0.21	400,718	0.904	0.037	1.4E-130	437,493	-0.038	0.001	4.2E-185	437,029	-1.655	0.088	4.0E-78
rs17615494	2	58,950,363	G	T	0.71	393,111	0.179	0.034	9.2E-08	429,146	-0.005	0.001	1.3E-04	428,697	0.104	0.080	1.9E-01
rs6728523	2	65,282,708	C	G	0.24	399,599	0.169	0.035	1.5E-06	436,276	-0.008	0.001	2.0E-11	435,814	0.085	0.084	3.1E-01
rs2280334	2	66,665,146	C	T	0.68	400,718	0.187	0.032	5.6E-09	437,493	-0.004	0.001	3.3E-04	437,029	0.031	0.076	6.9E-01
rs6720034	2	111,664,756	A	G	0.64	399,659	0.149	0.031	1.9E-06	436,345	0.001	0.001	0.26	435,882	0.101	0.074	1.7E-01
rs13396091	2	146,371,961	G	A	0.56	399,835	0.100	0.030	9.2E-04	436,524	-0.005	0.001	4.9E-06	436,061	-0.172	0.072	1.7E-02
rs5835988	2	165,501,927	TG	T	0.41	398,899	0.424	0.031	2.1E-43	435,476	-0.019	0.001	1.7E-69	435,010	-0.402	0.073	3.5E-08
rs72926946	2	203,477,868	C	A	0.71	399,648	0.273	0.033	1.1E-16	436,327	0.005	0.001	2.3E-05	435,866	0.544	0.078	3.7E-12
[b]rs78058190	2	219,699,999	G	A	0.97	369,221	1.080	0.088	8.3E-35	403,163	-0.042	0.003	3.8E-42	402,738	0.168	0.208	4.2E-01
rs2203452	2	227,094,758	A	G	0.35	400,602	0.626	0.031	2.1E-88	437,364	-0.021	0.001	1.6E-81	436,900	-0.025	0.075	7.4E-01
rs35240997	3	12,379,351	G	A	0.21	398,434	0.252	0.037	7.3E-12	434,999	-0.011	0.001	1.4E-16	434,540	0.235	0.087	7.1E-03
rs2044753	3	24,293,001	T	C	0.89	399,072	0.210	0.048	1.3E-05	435,688	-0.007	0.002	1.2E-05	435,226	-0.087	0.115	4.5E-01
rs6777217	3	36,979,042	G	A	0.56	399,114	0.148	0.030	1.1E-06	435,766	-0.005	0.001	7.8E-07	435,305	-0.135	0.072	6.2E-02
rs62246406	3	47,097,985	G	A	0.83	400,521	0.213	0.040	7.5E-08	437,274	-0.001	0.001	0.29	436,810	-0.159	0.094	9.1E-02

						HDL cholesterol (mg/dL)				Triglycerides (mg/dL)				LDL cholesterol (mg/dL)			
SNP	CHR	BP (hg19)	EA	NEA	EAF	N	BETA	SE	P-value	N	[a]BETA	[a]SE	[a]P-Value	N	BETA	SE	P-value
rs6808104	3	48,767,877	A	G	0.50	391,840	0.046	0.030	0.13	427,742	-0.001	0.001	0.21	427,284	0.086	0.072	2.4E-01
[d]rs111439884	3	50,024,038	A	C	0.50	364,876	0.327	0.032	2.7E-25	398,339	-0.004	0.001	1.7E-03	397,925	0.175	0.075	2.0E-02
rs11706108	3	52,372,366	C	T	0.19	400,080	0.336	0.038	1.4E-18	436,790	-0.015	0.001	7.6E-27	436,326	-0.119	0.091	1.9E-01
rs151105710	3	136,125,678	TTAAA	G	0.20	392,874	0.302	0.038	1.5E-15	428,937	-0.012	0.001	3.1E-20	428,486	-0.338	0.090	1.7E-04
rs3773910	3	152,171,870	G	C	0.26	396,945	0.135	0.034	8.9E-05	433,350	0.000	0.001	0.97	432,890	0.279	0.082	6.4E-04
rs9817452	3	156,795,414	T	G	0.38	398,010	0.258	0.031	1.0E-16	434,529	-0.010	0.001	5.8E-18	434,072	0.043	0.074	5.6E-01
[d]rs2268840	3	185,931,174	C	T	0.23	400,718	0.250	0.036	2.6E-12	437,493	-0.001	0.001	0.30	437,029	0.037	0.085	6.6E-01
rs11248051	4	858,332	C	T	0.91	400,718	0.214	0.052	4.0E-05	437,493	-0.007	0.002	1.0E-04	437,029	0.009	0.124	9.4E-01
[b]rs73243877	4	26,047,616	A	G	0.84	400,718	0.395	0.040	8.7E-23	437,493	-0.015	0.001	1.2E-24	437,029	-0.282	0.096	3.2E-03
rs1117816	4	69,349,018	C	A	0.24	400,178	0.238	0.035	1.0E-11	436,903	0.008	0.001	9.0E-10	436,439	0.750	0.083	1.9E-19
rs13133548	4	89,740,128	G	A	0.55	400,718	0.285	0.030	3.4E-21	437,493	-0.008	0.001	2.9E-14	437,029	-0.119	0.072	9.7E-02
rs12509976	4	100,517,324	T	C	0.04	400,672	0.380	0.077	8.4E-07	437,441	0.010	0.003	1.7E-04	436,977	0.570	0.184	1.9E-03
rs13107325	4	103,188,709	C	T	0.93	400,718	1.150	0.057	4.4E-90	437,493	-0.016	0.002	4.3E-15	437,029	0.812	0.136	2.4E-09
rs41278045	4	110,638,764	A	G	1.00	399,435	2.017	0.498	5.2E-05	436,097	-0.076	0.018	2.1E-05	435,634	-1.591	1.196	1.8E-01
rs17369400	4	154,224,048	A	G	0.90	400,718	0.133	0.049	6.7E-03	437,493	0.008	0.002	2.7E-06	437,029	0.544	0.117	3.4E-06
rs4691380	4	157,720,124	T	C	0.33	400,718	0.149	0.032	3.5E-06	437,493	-0.007	0.001	3.3E-10	437,029	0.015	0.076	8.4E-01
rs28499105	5	53,274,467	G	A	0.30	394,897	0.180	0.033	4.5E-08	431,170	-0.008	0.001	1.3E-11	430,723	0.163	0.078	3.7E-02
rs459193	5	55,806,751	A	G	0.26	400,718	0.380	0.035	3.0E-28	437,493	-0.017	0.001	2.2E-44	437,029	0.128	0.082	1.2E-01
rs6881956	5	72,926,514	G	A	0.71	398,091	0.119	0.033	3.6E-04	434,639	0.002	0.001	0.16	434,174	-0.067	0.079	4.0E-01
rs2307111	5	75,003,678	C	T	0.40	400,718	0.293	0.031	1.8E-21	437,493	-0.003	0.001	0.01	437,029	1.354	0.073	2.4E-76
rs1045241	5	118,729,286	T	C	0.27	394,973	0.231	0.034	1.4E-11	431,225	-0.011	0.001	4.4E-19	430,764	-0.057	0.081	4.8E-01
rs2434612	5	158,022,041	A	G	0.79	390,224	0.255	0.038	1.1E-11	426,032	-0.011	0.001	1.0E-16	425,585	-0.046	0.089	6.0E-01
rs7730898	5	170,459,675	G	A	0.27	400,088	0.147	0.034	1.5E-05	436,799	-0.003	0.001	0.04	436,336	0.027	0.080	7.3E-01
[d]rs9368830	6	34,668,635	T	C	0.66	400,276	0.350	0.032	5.7E-28	437,007	0.000	0.001	0.88	436,544	0.109	0.076	1.5E-01
[d]rs4130023	6	41,934,514	C	T	0.79	396,687	0.264	0.037	6.1E-13	433,086	-0.004	0.001	1.3E-03	432,621	-0.136	0.087	1.2E-01
rs998584	6	43,757,896	C	A	0.52	396,507	0.462	0.030	7.5E-53	432,896	-0.021	0.001	1.0E-85	432,437	-0.290	0.072	5.5E-05
rs2489629	6	127,476,717	C	T	0.45	400,630	0.267	0.030	1.0E-18	437,396	-0.012	0.001	4.5E-30	436,932	-0.490	0.072	9.4E-12
rs6925103	6	137,076,010	C	T	0.48	398,587	0.151	0.030	5.6E-07	435,173	-0.001	0.001	0.61	434,715	0.192	0.072	7.5E-03
rs199607859	6	139,835,418	T	G	0.59	396,110	0.304	0.031	5.2E-23	432,473	-0.015	0.001	6.5E-45	432,011	-0.270	0.073	2.2E-04
rs11751347	6	161,092,438	C	T	0.90	397,247	0.830	0.050	4.3E-61	433,697	-0.021	0.002	5.4E-31	433,234	0.662	0.120	3.3E-08
[d]rs79949326	7	6,461,310	T	C	0.26	399,140	0.383	0.034	9.8E-29	435,762	0.001	0.001	0.62	435,302	0.331	0.082	5.3E-05
rs3173615	7	12,269,417	C	G	0.58	400,718	0.161	0.030	1.2E-07	437,493	-0.005	0.001	4.1E-06	437,029	-0.104	0.072	1.5E-01
rs1917368	7	17,911,752	G	T	0.60	399,475	0.388	0.031	1.9E-36	436,140	-0.008	0.001	5.4E-14	435,681	0.172	0.073	1.8E-02
rs4722593	7	26,370,190	A	G	0.36	384,681	0.149	0.032	2.7E-06	419,944	-0.009	0.001	5.4E-14	419,493	-0.104	0.076	1.7E-01
rs55747707	7	73,037,366	A	G	0.20	398,723	0.551	0.038	1.1E-48	435,304	-0.055	0.001	1.5E-374	434,845	-0.110	0.089	2.2E-01
rs3211938	7	80,300,449	G	T	0.001	400,674	2.049	2.254	0.36	437,440	-0.008	0.082	0.92	436,976	-5.021	5.446	3.6E-01
rs6971365	7	130,432,481	T	C	0.71	398,643	0.465	0.033	7.9E-45	435,241	-0.017	0.001	7.6E-46	434,780	-0.318	0.079	5.3E-05
rs17173637	7	150,529,449	T	C	0.92	400,718	0.300	0.054	2.7E-08	437,493	-0.009	0.002	9.0E-07	437,029	-0.245	0.129	5.7E-02
rs2936512	8	6,599,005	C	T	0.68	394,755	0.159	0.032	8.3E-07	430,957	-0.003	0.001	0.02	430,500	0.369	0.077	1.5E-06
rs4841132	8	9,183,596	G	A	0.91	400,718	1.215	0.052	2.6E-119	437,493	0.003	0.002	0.08	437,029	1.518	0.125	3.8E-34
rs79407615	8	19,850,099	G	T	0.10	400,614	2.369	0.051	1.1E-478	437,377	-0.100	0.002	1.4E-674	436,912	-0.522	0.121	1.5E-05

						HDL cholesterol (mg/dL)				Triglycerides (mg/dL)				LDL cholesterol (mg/dL)			
**SNP**	**CHR**	**BP (hg19)**	**EA**	**NEA**	**EAF**	**N**	**BETA**	**SE**	**P-value**	**N**	**[a]BETA**	**[a]SE**	**[a]P-Value**	**N**	**BETA**	**SE**	**P-value**
rs10504474	8	71,260,460	G	A	0.90	400,557	0.312	0.050	5.2E-10	437,317	-0.004	0.002	0.05	436,852	-0.058	0.120	6.3E-01
[d]rs2721954	8	116,603,103	T	C	0.57	398,504	0.478	0.030	1.4E-55	435,109	-0.003	0.001	6.4E-03	434,648	-0.205	0.072	4.6E-03
rs2954038	8	126,507,389	A	C	0.70	400,718	0.556	0.033	1.3E-64	437,493	-0.045	0.001	7.1E-333	437,029	-1.530	0.078	7.9E-86
rs78123380	8	144,297,020	A	G	0.002	400,715	0.202	0.639	0.75	437,490	0.016	0.023	0.48	437,026	1.612	1.528	2.9E-01
[c]rs77375493	9	5,073,770	T	G	0.0001	400,233	0.572	1.765	0.75	436,967	-0.069	0.065	0.29	436,503	-4.189	4.313	3.3E-01
rs686030	9	15,304,782	A	C	0.86	399,977	0.786	0.043	4.8E-74	436,692	0.009	0.002	8.8E-09	436,229	0.356	0.103	5.4E-04
rs10963012	9	17,295,541	G	C	0.26	356,125	0.041	0.037	0.26	388,690	-0.002	0.001	0.10	388,266	-0.187	0.087	3.2E-02
rs4149307	9	107,589,744	T	C	0.16	400,278	0.957	0.042	3.6E-117	437,014	0.002	0.001	0.22	436,551	0.722	0.099	2.9E-13
rs4749779	10	8,576,206	G	A	0.27	399,266	0.074	0.034	0.03	435,908	-0.001	0.001	0.34	435,445	-0.057	0.080	0.47
[d]rs71515364	10	46,060,433	T	TAA	0.22	385,164	0.415	0.037	6.5E-29	420,418	0.000	0.001	0.82	419,968	0.161	0.088	6.9E-02
[d]rs1408579	10	101,912,194	T	C	0.49	400,631	0.198	0.030	4.7E-11	437,395	0.006	0.001	1.1E-08	436,931	0.365	0.072	3.3E-07
rs2792735	10	113,921,825	G	A	0.28	400,414	0.535	0.034	2.2E-57	437,168	-0.010	0.001	1.2E-15	436,705	0.594	0.080	9.7E-14
[d]rs72823013	10	115,786,233	A	G	0.12	398,711	0.443	0.045	1.9E-22	435,288	-0.007	0.002	4.1E-05	434,822	-0.731	0.108	1.4E-11
rs2901286	10	122,900,623	A	C	0.01	400,718	0.387	0.136	4.3E-03	437,493	-0.012	0.005	0.01	437,029	0.203	0.323	5.3E-01
rs6486121	11	13,355,770	C	T	0.37	399,727	0.071	0.031	0.02	436,433	-0.010	0.001	8.5E-18	435,968	-0.086	0.074	2.5E-01
rs150090666	11	14,865,399	T	C	0.001	400,274	5.449	0.566	6.7E-22	437,014	-0.204	0.020	7.0E-24	436,551	-3.920	1.354	3.8E-03
rs11434755	11	18,067,020	C	CA	0.58	394,345	0.050	0.031	0.10	430,542	-0.002	0.001	0.07	430,085	0.065	0.073	3.8E-01
rs1519480	11	27,675,712	T	C	0.67	398,567	0.212	0.032	4.5E-11	435,135	-0.007	0.001	1.7E-09	434,673	0.052	0.077	5.0E-01
rs75393320	11	47,266,471	C	G	0.15	399,646	0.763	0.042	5.3E-74	436,336	-0.014	0.001	1.9E-20	435,874	0.056	0.100	5.8E-01
rs174537	11	61,552,680	G	T	0.66	400,356	0.844	0.032	5.4E-157	437,096	-0.026	0.001	6.0E-118	436,633	1.104	0.075	1.0E-48
rs71468663	11	64,018,104	A	AC	0.96	399,677	0.747	0.072	3.6E-25	436,349	-0.035	0.003	1.7E-41	435,888	-0.453	0.171	8.2E-03
rs2306363	11	65,405,600	T	G	0.20	398,970	0.293	0.037	3.7E-15	435,586	-0.010	0.001	4.6E-15	435,125	0.558	0.089	2.9E-10
[b,d]rs11236564	11	75,567,227	G	A	0.92	398,426	0.544	0.054	1.5E-23	435,002	0.006	0.002	1.6E-03	434,543	0.218	0.129	9.3E-02
[d]rs689183	11	110,012,143	G	T	0.25	396,273	0.241	0.035	5.5E-12	432,665	-0.004	0.001	1.9E-03	432,211	0.009	0.083	9.1E-01
[c]rs138326449	11	116,701,354	A	G	0.001	399,975	12.781	0.451	8.3E-177	436,674	-0.553	0.016	1.1E-258	436,211	-4.632	1.077	1.7E-05
rs1945391	11	122,520,291	T	A	0.39	399,380	0.361	0.031	1.2E-31	436,040	0.002	0.001	0.08	435,578	0.427	0.073	6.3E-09
rs11054527	12	7,725,583	G	A	0.05	400,029	0.286	0.073	8.3E-05	436,735	-0.004	0.003	0.11	436,273	-0.292	0.173	9.2E-02
rs11045171	12	20,470,199	G	A	0.19	396,003	0.431	0.038	1.3E-29	432,364	-0.013	0.001	1.2E-21	431,904	-0.096	0.091	2.9E-01
rs10842708	12	26,474,867	A	G	0.75	398,510	0.217	0.035	6.2E-10	435,094	-0.008	0.001	5.8E-10	434,627	0.109	0.083	1.9E-01
rs4551851	12	33,459,554	G	A	0.49	396,646	0.079	0.030	9.2E-03	433,048	0.000	0.001	0.83	432,591	0.134	0.072	6.2E-02
rs1126930	12	49,399,132	G	C	0.96	400,718	0.260	0.081	1.4E-03	437,493	-0.012	0.003	3.7E-05	437,029	-0.158	0.194	4.1E-01
rs3809114	12	57,848,639	A	G	0.56	376,925	0.397	0.031	4.9E-37	411,597	-0.010	0.001	2.6E-18	411,155	-0.218	0.074	3.4E-03
rs2373459	12	101,873,956	T	C	0.67	398,470	0.148	0.032	3.8E-06	435,043	-0.002	0.001	0.06	434,578	0.001	0.076	9.9E-01
[d]rs7954144	12	110,015,893	G	A	0.52	400,611	0.344	0.030	2.5E-30	437,376	0.002	0.001	0.08	436,912	0.159	0.072	0.03
[c]rs4766578	12	111,904,371	A	T	0.51	400,556	0.351	0.030	1.6E-31	437,310	-0.004	0.001	3.5E-05	436,846	0.810	0.072	1.1E-29
rs10507274	12	117,160,976	C	T	0.06	400,718	0.320	0.061	1.8E-07	437,493	-0.003	0.002	0.13	437,029	-0.294	0.146	4.4E-02
[d]rs4759375	12	123,796,238	T	C	0.08	387,582	0.611	0.058	5.7E-26	423,139	0.001	0.002	0.54	422,698	0.392	0.138	4.5E-03
[d]rs10773112	12	125,338,529	T	C	0.72	400,718	0.513	0.033	5.4E-53	437,493	-0.001	0.001	0.45	437,029	0.149	0.080	6.2E-02
[d]rs13379043	14	74,250,126	C	T	0.28	376,410	0.296	0.035	1.7E-17	410,904	-0.003	0.001	0.02	410,476	-0.378	0.083	4.8E-06
[d]rs2494748	14	105,258,892	C	T	0.39	397,116	0.418	0.031	2.4E-41	433,550	-0.003	0.001	0.01	433,091	0.048	0.074	5.1E-01
[b]rs150844304	15	43,726,625	A	C	0.98	400,599	1.309	0.095	2.6E-43	437,367	-0.073	0.003	9.7E-103	436,903	-0.101	0.226	6.5E-01

SNP	CHR	BP (hg19)	EA	NEA	EAF	HDL cholesterol (mg/dL)				Triglycerides (mg/dL)				LDL cholesterol (mg/dL)			
						N	BETA	SE	P-value	N	[a]BETA	[a]SE	[a]P-Value	N	BETA	SE	P-value
rs139097404	15	43,933,941	T	C	0.98	398,986	1.281	0.101	8.2E-37	435,610	-0.074	0.004	3.6E-93	435,151	-0.005	0.241	9.8E-01
[b]rs2043082	15	58,674,308	A	G	0.35	399,253	1.464	0.031	5.1E-474	435,903	0.013	0.001	3.3E-33	435,441	0.542	0.075	4.3E-13
rs2245477	15	61,948,435	C	A	0.63	398,377	0.165	0.031	1.2E-07	434,918	-0.007	0.001	8.3E-10	434,459	-0.046	0.074	5.3E-01
[d]rs55703462	15	63,395,428	G	A	0.53	391,627	0.213	0.030	2.9E-12	427,560	-0.005	0.001	5.6E-07	427,104	0.168	0.072	2.1E-02
[c]rs139271800	15	90,214,777	G	A	0.001	400,192	1.681	0.618	6.5E-03	436,912	-0.078	0.022	3.8E-04	436,450	-1.169	1.458	4.2E-01
rs3751812	16	53,818,460	G	T	0.61	400,718	0.176	0.031	1.2E-08	437,493	-0.001	0.001	0.48	437,029	0.463	0.073	2.5E-10
rs821840	16	56,993,886	G	A	0.32	400,011	3.441	0.032	6.7E-2527	436,716	-0.017	0.001	1.6E-50	436,253	-1.109	0.076	1.2E-47
[d]rs56070533	16	67,942,320	A	G	0.12	400,596	0.891	0.047	6.2E-79	437,355	-0.007	0.002	2.9E-05	436,891	0.070	0.113	5.4E-01
[d]rs16958751	16	69,357,406	A	G	0.04	400,718	0.368	0.076	1.3E-06	437,493	-0.002	0.003	0.46	437,029	0.143	0.181	4.3E-01
rs5471	16	72,088,461	C	A	0.001	400,648	1.659	1.916	0.39	437,419	-0.204	0.069	3.0E-03	436,955	3.344	4.590	4.7E-01
rs2925979	16	81,534,790	C	T	0.70	400,718	0.549	0.033	5.4E-63	437,493	-0.017	0.001	1.3E-47	437,029	-0.135	0.078	8.4E-02
[d]rs12938449	17	486,821	C	A	0.81	399,930	0.247	0.038	8.4E-11	436,613	-0.007	0.001	6.3E-08	436,152	0.009	0.090	9.2E-01
[d]rs34879232	17	26,722,039	G	GA	0.45	393,083	0.201	0.030	4.8E-11	429,182	0.005	0.001	7.4E-07	428,721	-0.401	0.073	3.2E-08
[d]rs11078917	17	37,746,359	C	A	0.72	393,423	0.406	0.034	7.0E-33	429,565	-0.002	0.001	0.07	429,110	0.147	0.081	6.9E-02
rs72836561	17	41,926,126	C	T	0.97	400,718	2.261	0.086	1.1E-151	437,493	-0.072	0.003	1.2E-121	437,029	0.637	0.205	1.9E-03
rs56325564	17	45,766,771	A	G	0.48	390,132	0.109	0.031	3.6E-04	425,955	-0.002	0.001	0.12	425,496	0.887	0.073	2.2E-34
rs61676547	17	65,892,507	G	C	0.81	395,045	0.175	0.039	6.1E-06	431,267	-0.012	0.001	6.4E-18	430,808	-0.285	0.092	2.0E-03
rs12601079	17	76,400,329	A	G	0.60	389,598	0.437	0.031	1.0E-44	425,356	-0.010	0.001	2.7E-19	424,901	0.486	0.074	5.3E-11
rs1788783	18	21,161,134	T	C	0.50	397,378	0.217	0.030	7.0E-13	433,847	-0.008	0.001	1.6E-12	433,385	-0.193	0.072	7.4E-03
rs77960347	18	47,109,955	G	A	0.01	400,718	4.408	0.131	4.0E-249	437,493	0.018	0.005	1.5E-04	437,029	2.526	0.311	4.8E-16
rs12975319	19	3,414,088	G	A	0.70	392,249	0.185	0.033	2.5E-08	428,270	-0.002	0.001	0.049	427,815	-0.111	0.079	1.6E-01
rs56149994	19	7,242,261	C	T	0.79	394,895	0.210	0.038	2.4E-08	431,119	-0.009	0.001	6.5E-12	430,663	0.091	0.090	3.1E-01
rs116843064	19	8,429,323	A	G	0.02	400,718	3.020	0.109	2.4E-169	437,493	-0.117	0.004	1.1E-198	437,029	-0.081	0.259	7.6E-01
rs2278426	19	11,350,488	C	T	0.96	400,718	1.231	0.082	4.1E-51	437,493	0.022	0.003	6.8E-14	437,029	1.448	0.195	1.0E-13
[d]rs56121005	19	11,414,706	C	T	0.98	395,755	0.998	0.110	1.4E-19	432,066	0.016	0.004	4.6E-05	431,605	1.162	0.262	9.3E-06
rs748891192	19	33,940,662	G	[illegible]GGTG[illegible]	0.52	399,670	0.197	0.030	5.7E-11	436359	-0.005	0.001	8.3E-07	435896	0.136	0.072	0.06
rs429358	19	45,411,941	T	C	0.85	400,718	1.055	0.042	6.9E-142	437,493	-0.031	0.001	2.6E-99	437,029	-6.420	0.098	2.9E-921
rs61743199	19	50,161,091	A	G	0.93	400,718	0.315	0.058	5.9E-08	437,493	-0.003	0.002	0.13	437,029	0.349	0.138	0.01
[d]rs74256604	19	52,304,069	G	A	0.88	395,517	0.274	0.046	2.6E-09	431,783	-0.002	0.002	0.25	431,321	-0.044	0.110	6.9E-01
[d]rs380267	19	54,799,083	G	A	0.22	400,346	0.609	0.036	8.6E-64	437,081	-0.005	0.001	8.0E-05	436,617	0.313	0.086	2.8E-04
rs6120815	20	30,184,866	C	T	0.16	400,705	0.201	0.041	9.5E-07	437,479	-0.007	0.001	5.5E-06	437,015	0.057	0.098	5.6E-01
rs3746428	20	33,719,183	G	A	0.84	398,223	0.197	0.041	1.3E-06	434,778	-0.001	0.001	0.61	434,317	0.169	0.097	8.1E-02
rs1800961	20	43,042,364	C	T	0.97	400,718	1.956	0.086	3.5E-113	437,493	-0.006	0.003	0.04	437,029	2.019	0.206	1.2E-22
[d]rs2281279	20	46,290,250	C	T	0.30	400,718	0.215	0.033	5.0E-11	437,493	0.000	0.001	0.78	437,029	0.040	0.078	6.0E-01
rs856404	20	51,263,786	G	A	0.34	398,089	0.186	0.032	5.4E-09	434,622	-0.007	0.001	4.3E-09	434,159	-0.015	0.076	8.4E-01
rs2834707	21	36,343,552	C	T	0.64	398,680	0.149	0.031	1.7E-06	435,258	-0.002	0.001	0.07	434,793	0.026	0.074	7.3E-01
[d]rs235314	21	46,271,452	C	T	0.47	399,699	0.250	0.030	1.0E-16	436,366	0.002	0.001	0.02	435,903	-0.062	0.072	3.8E-01
[d]rs7444	22	21,976,934	T	C	0.80	400,427	0.445	0.038	1.7E-31	437,169	0.006	0.001	4.8E-06	436,705	0.369	0.091	4.6E-05
rs8142788	22	29,400,515	G	A	0.85	388,011	0.125	0.042	3.0E-03	423,622	-0.002	0.002	0.13	423,175	-0.040	0.100	6.9E-01
rs2899297	22	38,594,668	G	A	0.40	399,670	0.278	0.031	1.2E-19	436,353	-0.011	0.001	2.7E-25	435,895	-0.136	0.073	6.2E-02
rs2294915	22	44,340,904	C	T	0.77	398,589	0.185	0.036	2.2E-07	435,191	0.005	0.001	1.3E-04	434,728	0.242	0.085	4.4E-03

Table S3. Associations of 155 Previously Reported HDL Cholesterol SNPs with Risk of SARS-Cov-2 Infection.

EA, effect (HDL cholesterol raising) allele; NEA, non-effect allele.

Logistic regression analyses for risk of SARS-CoV-2 infection using unmatched hospital-based controls were adjusted for age, sex, PC1-10, and genotyping array.

Conditional logistic regression analyses for risk of SARS-CoV-2 infection using matched hospital-based controls were adjusted for PC1-10 and genotyping array.

[a]Excluded from tier 1 in MR analyses due to being either palindromic (rs4655268 and rs4766578) or missing association results for SARS-CoV-2 infection (rs77375493, rs138326449, rs139271800).

[b]Considered as tier 2 SNP in MR analyses (see Table S6).

					Cases vs. Unmatched Hospital-based Controls					Cases vs. Matched Hospital-based Controls				
**SNP**	**CHR**	**BP (hg19)**	**EA**	**NEA**	**N**	**OR**	**L95**	**U95**	**P-value**	**N**	**OR**	**L95**	**U95**	**P-value**
rs79598313	1	27,284,913	C	T	4,127	0.88	0.63	1.22	0.43	2156	0.90	0.60	1.37	0.63
rs3768321	1	40,035,928	G	T	4,112	1.08	0.95	1.22	0.26	2142	0.98	0.84	1.15	0.81
rs11207977	1	62,977,307	C	T	4,127	0.99	0.89	1.10	0.88	2157	1.12	0.97	1.28	0.12
[b]rs10874777	1	93,862,020	T	C	4,090	0.96	0.87	1.07	0.48	2123	0.97	0.85	1.11	0.68
rs2878349	1	107,549,245	G	A	4,125	1.04	0.93	1.16	0.50	2154	1.10	0.96	1.27	0.17
rs3832016	1	109,818,158	C	CT	4,124	1.06	0.93	1.20	0.39	2156	1.01	0.86	1.18	0.92
rs10494363	1	149,909,495	A	G	4,125	1.16	0.96	1.39	0.12	2157	1.21	0.97	1.51	0.10
[b]rs267738	1	150,940,625	G	T	4,127	0.99	0.87	1.11	0.81	2157	0.95	0.82	1.11	0.55
[b]rs4077194	1	178,533,832	G	T	4,120	0.96	0.87	1.06	0.41	2155	0.98	0.86	1.11	0.70
[b]rs61805076	1	182,154,990	T	C	4,109	1.03	0.93	1.15	0.57	2147	1.14	0.99	1.31	0.06
rs10911505	1	184,049,978	T	C	4,099	1.08	0.97	1.20	0.15	2140	1.02	0.89	1.17	0.76
[b]rs823114	1	205,719,532	A	G	4,119	1.11	1.00	1.23	0.04	2149	1.16	1.02	1.32	0.03
[a]rs4655268	1	214,992,980	C	G	3,955	0.99	0.89	1.09	0.79	2013	0.90	0.79	1.02	0.09
rs2066152	1	219,664,030	A	G	4,067	1.02	0.92	1.13	0.73	2112	0.98	0.86	1.12	0.75
rs2642438	1	220,970,028	G	A	4,127	1.07	0.96	1.20	0.23	2157	1.12	0.98	1.29	0.09
rs4846914	1	230,295,691	A	G	4,127	1.02	0.92	1.14	0.69	2157	1.09	0.95	1.24	0.22
[b]rs6710091	2	239,597	G	C	4,125	0.99	0.89	1.10	0.87	2156	0.96	0.84	1.09	0.54
[b]rs4850047	2	3,634,753	T	C	4,127	1.09	0.94	1.27	0.23	2157	0.89	0.73	1.08	0.23
rs676210	2	21,231,524	A	G	4,127	1.04	0.91	1.18	0.58	2157	0.95	0.81	1.11	0.50
rs17615494	2	58,950,363	G	T	4,045	0.97	0.87	1.09	0.61	2089	1.10	0.95	1.27	0.20
rs6728523	2	65,282,708	C	G	4,113	1.06	0.94	1.20	0.32	2144	1.04	0.89	1.20	0.63
rs2280334	2	66,665,146	C	T	4,127	1.00	0.89	1.11	0.96	2157	0.97	0.85	1.11	0.70
rs6720034	2	111,664,756	A	G	4,115	0.97	0.87	1.08	0.54	2155	1.09	0.95	1.25	0.22
rs13396091	2	146,371,961	G	A	4,117	1.04	0.94	1.16	0.44	2145	0.98	0.86	1.11	0.74
rs5835988	2	165,501,927	TG	T	4,111	1.05	0.94	1.16	0.38	2144	1.03	0.91	1.18	0.63
rs72926946	2	203,477,868	C	A	4,113	1.05	0.94	1.17	0.40	2147	0.94	0.82	1.08	0.39
rs78058190	2	219,699,999	G	A	3,847	1.03	0.77	1.38	0.82	1883	1.12	0.77	1.62	0.57
rs2203452	2	227,094,758	A	G	4,127	1.03	0.92	1.15	0.61	2157	1.01	0.88	1.16	0.88
rs35240997	3	12,379,351	G	A	4,110	1.11	0.98	1.26	0.09	2143	1.16	0.99	1.35	0.07

					Cases vs. Unmatched Hospital-based Controls					Cases vs. Matched Hospital-based Controls				
**SNP**	**CHR**	**BP (hg19)**	**EA**	**NEA**	**N**	**OR**	**L95**	**U95**	**P-value**	**N**	**OR**	**L95**	**U95**	**P-value**
rs2044753	3	24,293,001	T	C	4,105	1.01	0.86	1.18	0.92	2145	0.98	0.81	1.20	0.86
rs6777217	3	36,979,042	G	A	4,103	0.96	0.86	1.06	0.40	2134	0.91	0.80	1.04	0.16
rs62246406	3	47,097,985	G	A	4,122	0.91	0.79	1.04	0.17	2154	0.90	0.76	1.06	0.21
rs6808104	3	48,767,877	A	G	4,043	1.03	0.93	1.14	0.55	2075	1.10	0.97	1.24	0.16
[b]rs111439884	3	50,024,038	A	C	3,747	0.90	0.81	1.01	0.07	1848	0.90	0.78	1.03	0.12
rs11706108	3	52,372,366	C	T	4,123	0.89	0.78	1.02	0.09	2154	0.94	0.79	1.11	0.45
rs151105710	3	136,125,678	TTAA[illegible]	G	4,034	1.01	0.89	1.14	0.92	2068	0.91	0.78	1.07	0.27
rs3773910	3	152,171,870	G	C	4,092	1.12	1.00	1.26	0.05	2127	1.17	1.01	1.35	0.04
rs9817452	3	156,795,414	T	G	4,097	1.05	0.94	1.16	0.40	2125	0.94	0.83	1.08	0.40
[b]rs2268840	3	185,931,174	C	T	4,127	1.04	0.92	1.17	0.57	2157	1.05	0.91	1.22	0.52
rs11248051	4	858,332	C	T	4,127	1.25	1.03	1.51	0.02	2157	1.25	1.00	1.56	0.05
rs73243877	4	26,047,616	A	G	4,127	1.02	0.89	1.17	0.79	2157	1.00	0.84	1.19	1.00
rs1117816	4	69,349,018	C	A	4,121	0.95	0.85	1.07	0.43	2156	1.02	0.88	1.17	0.80
rs13133548	4	89,740,128	G	A	4,127	1.02	0.92	1.13	0.76	2157	0.89	0.79	1.01	0.08
rs12509976	4	100,517,324	T	C	4,127	0.79	0.60	1.04	0.10	2157	0.87	0.62	1.21	0.40
rs13107325	4	103,188,709	C	T	4,127	0.89	0.74	1.07	0.21	2157	0.73	0.58	0.93	0.01
rs41278045	4	110,638,764	A	G	4,112	3.26	0.41	25.70	0.26	2144	NA	NA	NA	NA
rs17369400	4	154,224,048	A	G	4,127	0.94	0.80	1.11	0.46	2157	0.85	0.70	1.04	0.12
rs4691380	4	157,720,124	T	C	4,127	0.95	0.85	1.06	0.38	2157	0.95	0.83	1.09	0.49
rs28499105	5	53,274,467	G	A	4,068	1.15	1.03	1.28	0.01	2098	1.05	0.91	1.20	0.52
rs459193	5	55,806,751	A	G	4,127	1.00	0.88	1.12	0.95	2157	0.97	0.84	1.13	0.73
rs6881956	5	72,926,514	G	A	4,092	1.01	0.90	1.13	0.90	2125	0.98	0.85	1.13	0.73
rs2307111	5	75,003,678	C	T	4,127	1.14	1.03	1.27	0.01	2157	1.09	0.96	1.24	0.20
rs1045241	5	118,729,286	T	C	4,072	1.11	0.99	1.25	0.07	2111	1.12	0.97	1.29	0.13
rs2434612	5	158,022,041	A	G	4,021	1.11	0.97	1.26	0.12	2057	1.04	0.88	1.22	0.69
rs7730898	5	170,459,675	G	A	4,119	1.01	0.90	1.14	0.86	2148	1.06	0.91	1.22	0.47
[b]rs9368830	6	34,668,635	T	C	4,125	0.97	0.87	1.09	0.64	2156	0.99	0.86	1.14	0.87
[b]rs4130023	6	41,934,514	C	T	4,090	0.88	0.78	1.00	0.05	2118	0.89	0.76	1.03	0.12
rs998584	6	43,757,896	C	A	4,090	1.06	0.96	1.18	0.23	2118	1.03	0.91	1.18	0.62
rs2489629	6	127,476,717	C	T	4,126	0.98	0.88	1.08	0.64	2156	0.97	0.86	1.10	0.67
rs6925103	6	137,076,010	C	T	4,110	0.95	0.86	1.05	0.31	2135	0.90	0.79	1.02	0.08
rs199607859	6	139,835,418	T	G	4,088	1.07	0.96	1.18	0.23	2130	1.03	0.91	1.17	0.65
rs11751347	6	161,092,438	C	T	4,094	1.03	0.87	1.22	0.75	2122	1.10	0.89	1.35	0.39
[b]rs79949326	7	6,461,310	T	C	4,113	0.93	0.83	1.05	0.24	2145	0.93	0.80	1.08	0.35
rs3173615	7	12,269,417	C	G	4,127	1.01	0.91	1.12	0.82	2157	1.02	0.90	1.16	0.76
rs1917368	7	17,911,752	G	T	4,120	0.98	0.89	1.09	0.78	2145	1.00	0.88	1.14	0.98
rs4722593	7	26,370,190	A	G	3,969	0.96	0.86	1.07	0.50	2027	0.97	0.84	1.12	0.68
rs55747707	7	73,037,366	A	G	4,104	0.91	0.80	1.03	0.15	2138	1.03	0.88	1.20	0.75

					Cases vs. Unmatched Hospital-based Controls					Cases vs. Matched Hospital-based Controls				
SNP	CHR	BP (hg19)	EA	NEA	N	OR	L95	U95	P-value	N	OR	L95	U95	P-value
rs3211938	7	80,300,449	G	T	4,126	NA	NA	NA	NA	2157	NA	NA	NA	NA
rs6971365	7	130,432,481	T	C	4,110	1.00	0.89	1.12	0.99	2150	1.04	0.91	1.19	0.57
rs17173637	7	150,529,449	T	C	4,127	1.02	0.84	1.22	0.87	2157	0.95	0.76	1.19	0.66
rs2936512	8	6,599,005	C	T	4,062	1.04	0.94	1.16	0.44	2110	0.95	0.83	1.09	0.48
rs4841132	8	9,183,596	G	A	4,127	1.09	0.91	1.30	0.36	2157	1.19	0.95	1.50	0.13
rs79407615	8	19,850,099	G	T	4,126	1.20	1.03	1.42	0.02	2154	1.11	0.90	1.37	0.34
rs10504474	8	71,260,460	G	A	4,125	0.94	0.79	1.12	0.49	2157	1.03	0.83	1.27	0.80
[b]rs2721954	8	116,603,103	T	C	4,112	1.04	0.94	1.15	0.47	2143	1.07	0.94	1.22	0.32
rs2954038	8	126,507,389	A	C	4,127	0.93	0.83	1.04	0.19	2157	0.90	0.79	1.04	0.15
rs78123380	8	144,297,020	A	G	4,127	NA	NA	NA	NA	2157	NA	NA	NA	NA
[a]rs77375493	9	5,073,770	T	G	4,121	NA	NA	NA	NA	2154	NA	NA	NA	NA
rs686030	9	15,304,782	A	C	4,122	1.04	0.90	1.21	0.58	2150	1.20	1.00	1.45	0.06
rs10963012	9	17,295,541	G	C	3,671	0.98	0.86	1.11	0.73	1769	0.95	0.81	1.12	0.55
rs4149307	9	107,589,744	T	C	4,122	1.06	0.92	1.22	0.42	2155	1.12	0.94	1.33	0.21
rs4749779	10	8,576,206	G	A	4,112	0.96	0.86	1.08	0.53	2145	1.03	0.90	1.18	0.70
[b]rs71515364	10	46,060,433	T	TAA	3,981	0.97	0.86	1.11	0.68	2008	0.95	0.81	1.12	0.55
[b]rs1408579	10	101,912,194	T	C	4,123	0.98	0.89	1.09	0.76	2151	1.05	0.92	1.19	0.46
rs2792735	10	113,921,825	G	A	4,127	0.99	0.88	1.11	0.87	2157	0.93	0.81	1.08	0.34
[b]rs72823013	10	115,786,233	A	G	4,110	0.99	0.85	1.16	0.89	2143	0.91	0.76	1.10	0.34
rs2901286	10	122,900,623	A	C	4,127	1.08	0.72	1.63	0.70	2157	1.38	0.82	2.33	0.23
rs6486121	11	13,355,770	C	T	4,118	1.08	0.97	1.20	0.16	2153	1.07	0.94	1.23	0.32
rs150090666	11	14,865,399	T	C	4,123	0.48	0.06	3.90	0.49	2156	1.00	0.09	10.99	1.00
rs11434755	11	18,067,020	C	CA	4,067	1.03	0.93	1.15	0.54	2106	1.14	1.00	1.30	0.05
rs1519480	11	27,675,712	T	C	4,100	1.01	0.90	1.12	0.89	2131	1.04	0.91	1.19	0.59
rs75393320	11	47,266,471	C	G	4,115	0.94	0.82	1.09	0.42	2145	0.95	0.80	1.14	0.60
rs174537	11	61,552,680	G	T	4,121	0.97	0.87	1.08	0.57	2154	0.93	0.81	1.06	0.29
rs71468663	11	64,018,104	A	AC	4,117	0.96	0.75	1.22	0.72	2155	0.95	0.71	1.28	0.75
rs2306363	11	65,405,600	T	G	4,114	0.89	0.78	1.01	0.08	2149	0.97	0.83	1.14	0.72
[b]rs11236564	11	75,567,227	G	A	4,108	1.00	0.83	1.21	1.00	2138	1.25	0.97	1.61	0.08
[b]rs689183	11	110,012,143	G	T	4,077	0.94	0.84	1.07	0.36	2110	1.08	0.93	1.25	0.32
[a]rs138326449	11	116,701,354	A	G	4,119	NA	NA	NA	NA	2148	NA	NA	NA	NA
rs1945391	11	122,520,291	T	A	4,115	0.97	0.88	1.08	0.62	2147	0.92	0.81	1.05	0.23
rs11054527	12	7,725,583	G	A	4,124	1.10	0.86	1.39	0.45	2152	1.20	0.89	1.62	0.23
rs11045171	12	20,470,199	G	A	4,076	1.05	0.93	1.20	0.42	2120	1.15	0.98	1.35	0.09
rs10842708	12	26,474,867	A	G	4,105	0.93	0.83	1.05	0.25	2131	0.95	0.82	1.10	0.47
rs4551851	12	33,459,554	G	A	4,088	1.00	0.90	1.11	0.97	2126	1.06	0.93	1.20	0.40
rs1126930	12	49,399,132	G	C	4,127	1.08	0.82	1.43	0.57	2157	1.32	0.92	1.89	0.13
rs3809114	12	57,848,639	A	G	3,888	0.91	0.81	1.01	0.07	1,922	1.04	0.91	1.19	0.58

SNP	CHR	BP (hg19)	EA	NEA	Cases vs. Unmatched Hospital-based Controls					Cases vs. Matched Hospital-based Controls				
					N	OR	L95	U95	P-value	N	OR	L95	U95	P-value
rs2373459	12	101,873,956	T	C	4,104	1.01	0.91	1.13	0.85	2,140	0.89	0.78	1.02	0.11
[b]rs7954144	12	110,015,893	G	A	4,125	1.03	0.93	1.14	0.53	2,156	0.96	0.85	1.09	0.53
[a]rs4766578	12	111,904,371	A	T	4,127	1.09	0.98	1.20	0.10	2,156	1.04	0.91	1.18	0.59
rs10507274	12	117,160,976	C	T	4,127	0.99	0.80	1.22	0.94	2,157	1.12	0.86	1.44	0.40
[b]rs4759375	12	123,796,238	T	C	3,995	0.93	0.76	1.14	0.46	2,037	0.97	0.77	1.24	0.83
[b]rs10773112	12	125,338,529	T	C	4,127	1.03	0.91	1.15	0.67	2,157	1.06	0.92	1.22	0.44
[b]rs13379043	14	74,250,126	C	T	3,866	0.89	0.79	1.01	0.07	1,931	0.91	0.78	1.06	0.20
[b]rs2494748	14	105,258,892	C	T	4,088	0.94	0.85	1.05	0.25	2,125	0.90	0.80	1.02	0.11
rs150844304	15	43,726,625	A	C	4,126	1.11	0.80	1.56	0.52	2,157	1.05	0.72	1.52	0.81
rs139097404	15	43,933,941	T	C	4,105	0.96	0.68	1.34	0.81	2,145	0.90	0.61	1.32	0.59
rs2043082	15	58,674,308	A	G	4,111	1.01	0.91	1.12	0.85	2,139	0.97	0.85	1.10	0.62
rs2245477	15	61,948,435	C	A	4,108	0.95	0.86	1.06	0.36	2,137	0.84	0.73	0.96	0.01
[b]rs55703462	15	63,395,428	G	A	4,060	1.01	0.91	1.12	0.92	2,089	0.99	0.87	1.12	0.84
[a]rs139271800	15	90,214,777	G	A	4,122	NA	NA	NA	NA	2,152	NA	NA	NA	NA
rs3751812	16	53,818,460	G	T	4,127	0.99	0.89	1.10	0.87	2,157	0.94	0.83	1.07	0.38
rs821840	16	56,993,886	G	A	4,119	1.00	0.90	1.11	0.99	2,146	1.01	0.88	1.15	0.93
[b]rs56070533	16	67,942,320	A	G	4,125	1.11	0.95	1.29	0.20	2,157	1.07	0.88	1.29	0.53
[b]rs16958751	16	69,357,406	A	G	4,127	0.74	0.56	0.97	0.03	2,157	0.60	0.42	0.85	3.7E-03
rs5471	16	72,088,461	C	A	4,127	NA	NA	NA	NA	2,155	NA	NA	NA	NA
rs2925979	16	81,534,790	C	T	4,127	1.05	0.94	1.18	0.36	2,157	0.99	0.86	1.14	0.92
[b]rs12938449	17	486,821	C	A	4,117	1.08	0.95	1.24	0.22	2,152	1.01	0.86	1.19	0.89
[b]rs34879232	17	26,722,039	G	GA	4,044	0.90	0.81	1.00	0.04	2,079	0.93	0.81	1.06	0.25
[b]rs11078917	17	37,746,359	C	A	4,058	1.01	0.90	1.14	0.81	2,105	1.04	0.90	1.20	0.58
rs72836561	17	41,926,126	C	T	4,127	0.90	0.67	1.21	0.49	2,157	0.74	0.52	1.05	0.09
rs56325564	17	45,766,771	A	G	4,015	0.99	0.89	1.10	0.88	2,061	0.99	0.87	1.13	0.91
rs61676547	17	65,892,507	G	C	4,079	1.09	0.96	1.25	0.18	2,119	1.05	0.89	1.23	0.57
rs12601079	17	76,400,329	A	G	4,002	1.08	0.97	1.21	0.15	2,061	1.06	0.93	1.21	0.39
rs1788783	18	21,161,134	T	C	4,092	0.93	0.84	1.03	0.18	2,129	0.91	0.80	1.03	0.13
rs77960347	18	47,109,955	G	A	4,127	1.13	0.73	1.74	0.59	2,157	1.07	0.63	1.81	0.81
rs12975319	19	3,414,088	G	A	4,037	0.98	0.88	1.10	0.72	2,077	1.00	0.87	1.15	0.98
rs56149994	19	7,242,261	C	T	4,077	1.03	0.91	1.17	0.67	2,112	1.14	0.97	1.34	0.11
rs116843064	19	8,429,323	A	G	4,127	1.23	0.87	1.73	0.24	2,157	1.06	0.70	1.62	0.78
rs2278426	19	11,350,488	C	T	4,127	1.14	0.86	1.49	0.37	2,157	1.05	0.75	1.45	0.79
[b]rs56121005	19	11,414,706	C	T	4,078	1.34	0.92	1.96	0.13	2,114	1.21	0.78	1.88	0.39
rs748891192	19	33,940,662	G	GGTGTC	4,121	1.03	0.87	1.21	0.76	2,145	0.97	0.85	1.10	0.63
rs429358	19	45,411,941	T	C	4,127	0.79	0.69	0.90	4.6E-04	2,157	0.84	0.71	0.99	0.03
rs61743199	19	50,161,091	A	G	4,127	1.12	0.92	1.37	0.26	2,157	1.10	0.86	1.41	0.45
[b]rs74256604	19	52,304,069	G	A	4,081	1.01	0.86	1.19	0.90	2,109	1.01	0.83	1.23	0.94

SNP	CHR	BP (hg19)	EA	NEA	Cases vs. Unmatched Hospital-based Controls					Cases vs. Matched Hospital-based Controls				
					N	OR	L95	U95	P-value	N	OR	L95	U95	P-value
[b]rs380267	19	54,799,083	G	A	4,120	0.93	0.83	1.06	0.28	2,150	0.92	0.78	1.07	0.28
rs6120815	20	30,184,866	C	T	4,127	0.93	0.81	1.07	0.33	2,157	0.93	0.78	1.11	0.43
rs3746428	20	33,719,183	G	A	4,099	1.03	0.89	1.19	0.69	2,134	1.05	0.88	1.26	0.57
rs1800961	20	43,042,364	C	T	4,127	1.12	0.82	1.53	0.46	2,157	1.15	0.80	1.66	0.45
[b]rs2281279	20	46,290,250	C	T	4,127	1.06	0.95	1.18	0.31	2,157	1.00	0.87	1.14	0.96
rs856404	20	51,263,786	G	A	4,107	1.06	0.95	1.18	0.31	2,135	0.98	0.85	1.12	0.75
rs2834707	21	36,343,552	C	T	4,111	1.02	0.91	1.13	0.78	2,134	1.05	0.92	1.20	0.48
[b]rs235314	21	46,271,452	C	T	4,119	1.01	0.91	1.12	0.86	2,150	0.96	0.84	1.09	0.53
[b]rs7444	22	21,976,934	T	C	4,122	1.05	0.92	1.19	0.50	2,147	0.99	0.84	1.16	0.86
rs8142788	22	29,400,515	G	A	3,989	0.93	0.81	1.07	0.34	2,035	0.90	0.75	1.07	0.24
rs2899297	22	38,594,668	G	A	4,115	0.97	0.87	1.08	0.56	2,142	0.98	0.86	1.11	0.77
rs2294915	22	44,340,904	C	T	4,109	1.06	0.94	1.20	0.34	2,136	1.04	0.90	1.21	0.60

Table S4. Association of ApoE4 Alleles with Severe SARS-Cov-2 Infection and Mortality from COVID-19 After Removal of Patients Diagnosed with Dementia or Alzheimer's Disease Prior to January 2020.

Data are shown as odds ratios (OR) and 95% confidence intervals (CI).
*Only includes subjects with complete data on HDL cholesterol levels.
[a]Logistic regression analysis: Model 1 was adjusted for age, sex, obesity, hypertension, type 2 diabetes, CAD, BMI, education, smoking, PC1-10, and genotyping array. Model 2 was adjusted for the same variables as model 1 with additional adjustment for HDL cholesterol levels.
[b]Conditional logistic regression analysis: Model 1 was adjusted for BMI, education, smoking, PC1-10, and genotyping array.
Model 2 was adjusted for the same variables as model 1 with additional adjustment for serum HDL cholesterol levels.
[c]P-values are for comparisons between E4/E4 vs. E3/E3 groups.

	ApoE Genotypes			
**Comparison Groups***	**E3/E3**	**E3/E4**	**E4/E4**	**[c]P-value**
**[a]Cases vs. Unmatched Hospital-based Controls (n)**	492/1,719	197/696	34/68	
Model 1	1	1.04 (0.86 - 1.26)	1.86 (1.21 - 2.86)	4.9E-03
Model 2	1	1.03 (0.85 - 1.25)	1.83 (1.19 - 2.83)	5.7E-03
**[b]Cases vs. Matched Hospital-based Controls (n)**	324/625	118/236	20/21	
Model 1	1	1.09 (0.82 - 1.46)	2.15 (1.04 - 4.41)	0.04
Model 2	1	1.10 (0.83 - 1.48)	2.13 (1.04 - 4.37)	0.04
**[a]Mortality among Cases (Yes/No)**	209/283	87/110	19/15	
Model 1	1	1.17 (0.79 - 1.75)	2.52 (1.12 - 5.69)	0.03
Model 2	1	1.16 (0.78 - 1.73)	2.52 (1.12 - 5.70)	0.03

Table S5. Cumulative Genetic Association of HDL Cholesterol-Raising Alleles with Risk of Severe SARS-Cov-2 Infection.

Number of cases and controls for each comparison group are shown in parentheses.

Data are shown as odds ratios (OR) and 95% confidence intervals (CI) for a 1-unit increase in genetic risk scores (GRS) constructed for HDL cholesterol levels with 155 previously reported SNPs.

Unweighted GRS were based on summing the number of HDL cholesterol-raising alleles for each subject used from the UK Biobank.

Weighted GRS were constructed by multiplying the number of HDL cholesterol-raising alleles carried by each subject from the UK Biobank by the respective weight (beta) for each variant as shown in Table S2 or the weight taken from a previously reported GWAS for HDL levels (Klarin et al. 2018; PMID 30275531).

[a]Logistic regression adjusted for age, sex, obesity, hypertension, type 2 diabetes, CAD, BMI, education, smoking, PC1-10, and genotyping array.

[b]Conditional logistic regression adjusted for BMI, education, smoking, PC1-10, and genotyping array.

		GRS for HDL cholesterol (range)					
		Unweighted (128-183)		Weighted (61.8-95.8)		Klarin et al. Weighted (5.28-8.23)	
**Comparison Groups**	**n**	**OR (95% CI)**	**P-value**	**OR (95% CI)**	**P-value**	**OR (95% CI)**	**P-value**
**[a]Cases vs. Unmatched Hospital-based Controls**	1,117/3,544	1.00 (0.99 - 1.01)	0.47	1.01 (0.99 - 1.02)	0.50	1.10 (0.87 - 1.38)	0.44
**[b]Cases vs. Matched Hospital-based Controls**	719/1,438	1.00 (0.99 - 1.01)	0.82	1.00 (0.97 - 1.02)	0.69	0.95 (0.70 - 1.29)	0.75
**[a]Mortality Among Cases (Yes/No)**	526/591	1.00 (0.98 - 1.02)	0.73	0.99 (0.96 - 1.02)	0.54	0.84 (0.53 - 1.33)	0.45

Table S6. Mendelian Randomization Analyses with HDL Cholesterol-raising Alleles and Risk of Severe SARS-Cov-2 Infection.

Data are shown as odds ratios (OR) and 95% confidence intervals (CI) for various combinations of HDL cholesterol-associated SNPs used as instrumental variables with HDL cholesterol as the exposures.

[a]Betas for HDL cholesterol levels and SARS-CoV-2 infection based on white European ancestry subjects and cases vs. unmatched hospital-based controls in the UK Biobank, respectively.

[b]Betas for HDL cholesterol levels and SARS-CoV-2 infection based on white European ancestry subjects and cases vs. matched hospital-based controls in the UK Biobank, respectively.

[c]Betas for HDL cholesterol levels and SARS-CoV-2 infection taken from Klarin et al. 2018 (PMID: 30275531) and Ellinghaus et al. 2020 (PMID: 32558485), respectively.

[d]Tier 1 did not include palindromic variants where both alleles had similar frequencies or SNPs with missing association results for HDL cholesterol levels or risk of SARS-CoV-2 infection.

[d]Tier 2: Only included variants associated with HDL cholesterol, but not LDL cholesterol or triglyceride, levels at the genome-wide significance threshold ($P=5.0x10^{8}$) .

[e]Exposure variables in multivariate MR (MVMR) analyses were HDL cholesterol, LDL cholesterol, and triglyceride levels but only results for HDL cholesterol are shown.

		[a]Analysis I			[b]Analysis II			[c]Analysis III		
**MR Method**	**[d]Tier**	**# of SNPs**	**OR (95% CI)**	**P-value**	**# of SNPs**	**OR (95% CI)**	**P-value**	**# of SNPs**	**OR (95% CI)**	**P-value**
MR Egger	1	147	1.00 (0.97 - 1.03)	0.95	146	0.99 (0.96 - 1.02)	0.61	144	0.91 (0.57 - 1.47)	0.71
Weighted median		147	1.00 (0.97 - 1.03)	0.99	146	1.00 (0.96 - 1.04)	0.95	144	0.87 (0.63 - 1.21)	0.42
Inverse variance weighted		147	1.00 (0.98 - 1.01)	0.64	146	0.98 (0.95 - 1.00)	0.09	144	0.96 (0.56 - 1.64)	0.88
[e]Inverse variance weighted - MVMR		147	1.00 (0.97 - 1.02)	0.79	146	0.99 (0.96 - 1.02)	0.52	144	N/A	N/A
MR Egger	2	35	1.06 (0.89 - 1.26)	0.53	35	1.02 (0.80 - 1.29)	0.89	35	1.60 (0.20 - 13.1)	0.66
Weighted median		35	0.97 (0.90 - 1.06)	0.54	35	0.92 (0.83 - 1.02)	0.13	35	1.07 (0.44 - 2.62)	0.88
Inverse variance weighted		35	0.96 (0.90 - 1.02)	0.19	35	0.93 (0.86 - 1.02)	0.11	35	1.06 (0.29 - 3.84)	0.93
[e]Inverse variance weighted - MVMR		33	0.95 (0.88 - 1.02)	0.17	33	0.95 (0.86 - 1.05)	0.33	35	N/A	N/A
